# Analysis of the Aroma Chemical Composition of Commonly Planted Kiwifruit Cultivars in China

**DOI:** 10.3390/foods10071645

**Published:** 2021-07-16

**Authors:** Tian Lan, Chenxu Gao, Quyu Yuan, Jiaqi Wang, Hexin Zhang, Xiangyu Sun, Yushan Lei, Tingting Ma

**Affiliations:** 1 College of Food Science and Engineering, Northwest A&F University, Yangling 712100, China; lt771451884@nwafu.edu.cn (T.L.); 2018013406@nwafu.edu.cn (C.G.); y23933@nwafu.edu.cn (Q.Y.); zhanghexin1217@nwafu.edu.cn (H.Z.); 2 College of Enology, Northwest A&F University, Yangling 712100, China; hello-wjq@nwafu.edu.cn (J.W.); sunxiangyu@nwafu.edu.cn (X.S.); 3 Shaanxi Rural Science and Technology Development Center, Xi’an 710054, China; leiyushanwz@sina.com

**Keywords:** kiwifruit, chemical compositions, E-nose, GC-MS, flesh color, species

## Abstract

The aroma chemical composition of commonly planted kiwifruit cultivars in China was analyzed. The combination of 2-octanone with 3-octanone was the most suitable dual internal standard for quantitative analysis in GC-MS. A total of 172 aroma components in 23 kiwifruit cultivars were detected, and ethyl butanoate, (E)-2-hexen-1-ol, and (E)-2-hexenal could be considered the core aroma components in kiwifruit, but still need further confirmation using Sensomics. E-nose could effectively distinguish different cultivars of kiwifruit. Clustering based on GC-MS and E-nose results tends to be consistent and demonstrate a certain degree of similarity. Kiwifruit cultivars with different flesh colors cannot be effectively distinguished by their aroma chemical compositions. Different species of kiwifruit can be distinguished to some extent by their aroma chemical compositions, but the effect was not satisfactory. These results could prove valuable in the breeding, planting, and marketing of kiwifruits.

## 1. Introduction

Kiwifruit (*Actinidia chinensis* Planch) is one of the most commercially valuable fruit species [1]. With the development of the kiwifruit industry and the progress of breeding technology based on Hayward and Hort16A, some countries have bred a number of new kiwifruit cultivars and promoted their cultivation. A large number of new cultivars of kiwifruit have been bred and widely cultivated also in China, the country of origin of kiwifruit, as a consequence of the rise in kiwifruit industry. Since 2000, the area and output of kiwifruit have been the largest in the world (Date from FAO: 1961–2018).

The genus *Actinidia* consists of more than 50 species. At present, *A. chinensis* Planch. var. *chinensis* (*A. chinensis*) and *A. chinensis* var. *deliciosa* A. Chevalier (*A*. *deliciosa*) are the most commercially valuable species [2,3]. Currently, Chinese kiwifruit are mainly sold in the domestic market, and the prices are generally low. The price is usually one-fifth to one-third of imported kiwifruit. Additionally, there is still a large demand for kiwifruit imports in the domestic fresh fruit market, and the price is much higher than that of domestic kiwifruit. In addition, Chinese kiwifruit exports are also small compared to imports [4]. Chinese kiwifruit production accounted for 70% of the world’s total production in 2019, but the import volume is still much higher than the export volume. In contrast, nearly 95% of kiwifruit produced in New Zealand are exported. Many factors have contributed to the above situation. Many factors have led to the above situation. One of the most important reasons is that the product characteristics of these new cultivars of kiwifruit, such as nutritional composition, are not clear, so the consumers’ degree of understanding and trust is poor. Conversely, New Zealand’s disclosure of the nutritional and functional properties of new commercial cultivars [5,6,7,8] promoted its successful commercialization and increased consumer acceptance. Therefore, fully studying the product characteristics and other indicators of the new cultivars of kiwifruit bred in China will be expected to provide help for the commercialization of new kiwifruit cultivars in China and provide more resources for consumers to purchase kiwifruit.

In recent years, new kiwifruit cultivars widely accepted by consumers have shown high-quality flavor characteristics, indicating that taste (sweetness and acidity) and aroma are the basic characteristics affecting the fruit market and consumer acceptance. Aroma characteristics are among the most important organoleptic characteristics for kiwifruit quality and are the main concern to consumers. Thus, research on the aroma of kiwifruit has gradually gained attention, and an increasing number of researchers have studied the aroma characteristics of kiwifruit.

At present, research on the aroma characteristics and composition of kiwifruit is mainly based on GC-MS [9], E-nose [9], and GC-O (gas chromatography-olfactometry) [10]. Du et al. [9] traced the internal quality and aroma of Hongyang during ripening by means of GC-MS and E-nose. The unique aroma components of different species of kiwifruit were summarized by studying the aromas of Cuiyu (*A. deliciosa*), Jintao (*A. chinensis*), and Tuguomaohua (*A. eriantha*) varieties, but with only one sample per species [11]. The aroma components of kiwifruit “Jecy Green” [12], the aromas of Hayward and Hort16A [13], and the aroma characteristics of four yellow-fleshed kiwifruits and two green-fleshed kiwifruits (both *A. chinensis*) [14] were also analyzed or reviewed. Overall, in these previous studies, the aroma of kiwifruit is understood in some detail, yet further research is still required. Most of the current research on volatiles in kiwifruit has addressed determination of the volatile organic compound content according to the physiological development and storage of the fruit, while only a few studies have focused on the qualitative and semiquantitative variation of the volatile composition in kiwifruit cultivars [14]. Fewer or single cultivars and species have been selected, and single analysis methods have been used for research in most studies. Therefore, these studies lack universality. At the same time, there are very few reports on the aroma characteristics of new cultivars developed in China.

The current study was performed with 20 Chinese independently bred new cultivars, 2 introduced cultivars (Hayward and Hort16A), and using an imported cultivar Sungold (New Zealand) as control. The aroma chemical composition of kiwifruit samples was analyzed through GC-MS and E-nose analysis. The aim of this paper is (1) to clarify the aroma chemical compositions of 20 Chinese independently bred new cultivars and compare them with imported and introduced cultivars; (2) to compare and analyze the aroma chemical compositions of kiwifruit of different species (*A. deliciosa* and *A. chinensis*) and different flesh colors based on the largest sample size to date, and (3) to classify the aroma chemical compositions of kiwifruit by LDA and clustering analysis and to lay the foundation for future research on kiwifruit aroma. The research will provide a reference for kiwifruit breeding, planting, sales, and purchase.

## 2. Materials and Methods

### 2.1. Chemicals and Reagents

Sodium chloride was of analytical grade and purchased from GuangXi XiLong Chemical Co. Ltd. (Guangxi, China). Internal standards included (S)-(+)-2-octanol, 3-octanol, adonitol, cyclohexanone, 2-octanone, 3-octanone, 2-nonane, 3-nonane, octyl acetate, ethyl caprylate, and triacetine, purchased from Shanghai Yuanye Bio-Technology Co. Ltd. (Shanghai, China) and Aladdin (Shanghai, China), and all are of chromatographic grade.

### 2.2. Sample Collection

**Figure 1:**
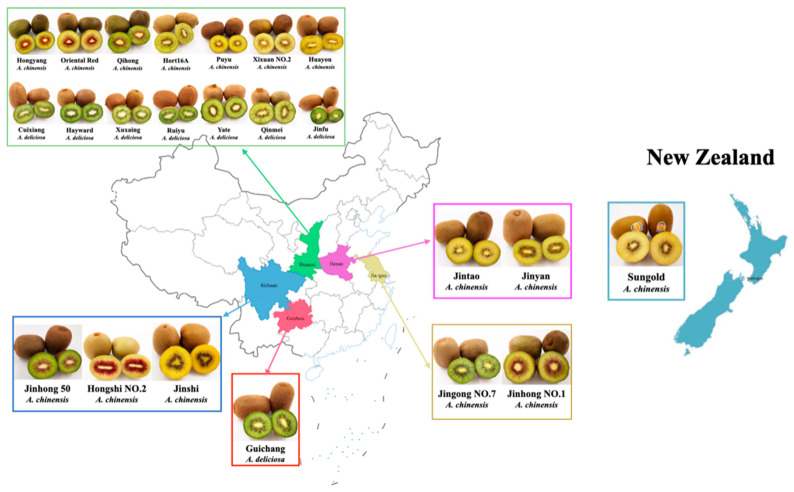
Origins, cultivars, flesh colors, and species of kiwifruit.

Fresh kiwifruit that had reached commercial maturity were bought from producers and fresh fruit markets. A total of 23 cultivars were purchased: 20 new breeds from five different provinces (Shaanxi, Sichuan, Guizhou, Henan, and Jiangsu) in China; 2 introduced cultivars (Hayward and Hort16A) from Shaanxi, China; and 1 imported cultivar, Sungold, from New Zealand. All the materials harvested in 2019 were at commercial maturity with uniform size, and their hardness was between 4.0 and 7.0 N/cm^2^. Relevant sample information, including origin, species, and flesh color, is shown in Figure 1 and Table S1.

### 2.3. HS-SPME/GC-MS Analysis

Kiwifruit samples (20 kiwifruits per sample) were peeled and homogenized to obtain fruit pulp. Five milliliters of each kiwifruit pulp was transferred to 20 mL headspace vials. Then, 1.5 g of sodium chloride and 120 μL of the internal standard mixture were added to kiwifruit pulp. The determination and selection of the internal standard are shown in Table S2 and Table 1. The concentration of all internal standards was 100 μL/L.

The volatile components in kiwifruit samples were analyzed by HS-SPME (headspace solid-phase microextraction) combined with GC-MS. The methods were based on previous studies [15,16] with minor modifications. Samples were equilibrated at 45 °C for 30 min, and the volatile compounds were extracted from the headspace to the SPME fiber (50/30 μm, DVB/CAR/PDMS, Supelco, Bellefonte, USA) for 30 min. Then, the SPME fiber was injected into the injection port of the GC-MS system and desorbed at 250 °C for 2 min. A GC-MS-QP2010 (Shimadzu, Kyoto, Japan) was employed for qualitative and quantitative analysis of the volatile compounds. A fused silica capillary column (DB-1MS; 30 m × 250 μm × 0.25 μm) and helium gas (1.93 mL/min) were used for detection. The initial temperature was 40 °C, maintained for 3 min, the temperature rose to 120 °C at a speed of 4 °C/min, then rose at a rate of 6 °C/min to 240 °C and was maintained for 9 min. The mass spectrometry (MS) conditions were as follows: electron impact ionization (EI), ion source temperature was 230 °C, ionization energy was 70 eV, and the scanning range of MS was *m*/*z* 35–500 in full-scan mode.

According to the NIST 14 mass spectrometry database, each volatile component was identified by matching degree, retention time, and retention index (RI). Volatile components with matching degrees greater than 85% were selected as effective aroma components. Then, the internal quantification standards in Table 1 were used to quantify the volatile components in the kiwifruit samples. Meanwhile, internal verification standards were used to verify the quantitative results. The results showed that the relative standard deviation (% RSD) between the internal quantification standard and the internal verification standard was less than 15%. All tests were performed in triplicate.

### 2.4. E-Nose Analysis

This experiment was performed using PEN 3 (Airsense Analytics, Schwerin, Germany). The E-nose has 10 metal oxide semiconductors. The 10 sensors are W1C (benzene and aromatic compounds), W5S (broad-range sensitivity, very sensitive to nitrogen oxides), W3C (ammonia, sensitive to aromatic compounds), W6S (mainly hydrogen, selectively), W5C (alkane, aromatic compounds), W1S (sensitive to methane, broad range.), W1W (sensitive to many sulfur organic compounds and terpenes.), W2S (alcohol, sensitive to aromatic compounds with broad range, similar to No. 6.), W2W (aromatic compounds and sulfur organic compounds) and W3S (reacts at high concentrations, very sensitive to several compounds) [17].

Kiwifruit samples (5 kiwifruits per sample) were washed, peeled, and homogenized. Five milliliters of kiwifruit pulp samples were placed in 20 mL vials, testing started after equilibrating at 25 °C for 10 min, and each kiwifruit sample was determined at least 10 times. The specific parameters of E-nose detection were as follows: the carrier gas velocity 300 mL/min, the detection time duration 60–80 s, and the cleaning time 300 s. This method was slightly modified based on the research of Ma et al. [15].

### 2.5. Statistical Analysis

In this study, Excel 16.41 was used to integrate and summarize the data of GC-MS and E-nose. SPSS 26.0 (IBM, Armonk, NY, USA) was used to perform LDA data analysis and principal component analysis (PCA) on GC-MS and E-nose data. The systematic clustering analysis was performed based on GC-MS and E-nose data through TBtools (https://github.com/CJ-Chen/TBtools/releases accessed on: 7 March 2020) and SPSS 26.0. Specifically, the standardized data were used to perform systematic cluster analysis based on different kiwifruit varieties, and the clustering method involved was intergroup connection. TBtools was used to draw heat maps and Venn diagrams.

## 3. Results and Discussion

### 3.1. HS-SPME-GC-MS Analysis

#### 3.1.1. The Determination of Internal Standards

The internal standards fulfilled three criteria: (1) their retention times were free of possible coelutions, (2) they were robust and stable for use in a long sample sequence, and (3) they were not initially present in the samples [18]. Based on previous studies, this study tested 11 standard substances to screen out universal internal standards for qualitative and quantitative analysis of HS-SPME-GC-MS (see Table S2 for details).

The results indicated that six standard substances can be used as internal standards for kiwifruit samples, namely, (S)-(+)-2-octanol, 3-octanol, 2-octanone, 3-octanone, 2-nonane, and 3-nonane. The four standard substances, adonitol, octyl acetate, ethyl caprylate, and triacetin, were not detected after the addition, which may be due to the reaction with the components in the kiwifruit samples or coelution. The detection results for cyclohexanone were significantly different from those obtained by universal internal standards, so it was not suitable for this experiment. After screening, six universal internal standards were used to form four groups of double internal standards for the qualitative and quantitative analysis of volatile components in GC-MS. Among them, 2-octanone and 3-octanone have the highest universal applicability, the smallest error (0.22–4.89% RSD), and can be used for the determination of 18 kiwifruit samples, 2-octanone and (S)-(+)-2-octanol can be used for the determination of 3 kiwifruit samples, while 2-nonanone, 3-nonanone, 3-octanol and 3-octanone can only be used for the determination of one kiwifruit sample. See Table 1 for internal standard selection and errors of different kiwifruit samples.

#### 3.1.2. HS-SPME-GC-MS Analysis of Volatile Components in Kiwifruit Samples

This research used HS-SPME-GC-MS for qualitative and quantitative analysis of volatile components in 23 kiwifruit samples. A total of 172 volatile compounds were detected by HS-SPME-GC-MS (see Table 2 for details). They were divided into nine categories according to functional groups, including 37 esters, 17 alcohols, 13 aldehydes, 16 ketones, 4 acids, 20 alkanes, 40 alkenes, 16 aromatic hydrocarbons and their homologues, and 9 other components.

Firstly, the volatiles contained in 20 or more kiwifruit samples were defined as the main volatiles in kiwifruit. There were 16 primary volatiles in total, namely, ethyl acetate, n-propyl acetate, ethyl butanoate, hexyl formate, ethyl hexanoate, diisobutyl phthalate, ethanol, (E)-2-hexen-1-ol, (E)-2-hexenal, styrene, D-limonene, 3-isopropylide-6-methyl-1-cyclohexene, o-xylene, 3,5-bis(1,1-dimethylethyl)-phenol, ammonium carbamate, and methoxy-phenyl-oxime. Esters are the most abundant volatiles, with six kinds.

Secondly, volatiles commonly contained in samples of less than 20 cultivars and more than 12 cultivars of kiwifruit were defined as secondary volatiles. There were 21 secondary volatiles, including 2 esters, 3 alcohols, 4 aldehydes, 1 ketone, 2 alkanes, 4 alkenes, 2 aromatic hydrocarbons and their homologs, and 3 other compounds. 

Third, volatile substances contained in only one kiwifruit sample were defined as unique volatiles. There were 74 unique volatiles in 23 kiwifruit samples.

Among the 23 kiwifruit cultivars in this study, there were 37 volatile components in Sungold, including 13 primary volatiles, 10 secondary volatiles, and 3 unique volatiles. There were 33 volatile components in Hayward, including 15 primary volatiles, 12 secondary volatiles, and no unique volatiles. Additionally, there were 32 volatile components in Hort16A, including 15 primary volatiles, 11 secondary volatiles, and one unique volatile. The number of volatile components in new Chinese breeds ranged from 37 to 79, greater than the numbers in imported and introduced cultivars. 

#### 3.1.3. Qualitative and Quantitative Analysis of Volatile Components in Kiwifruit Samples

To obtain a more detailed understanding of the volatile components in kiwifruit samples, the nine categories of volatile components were divided into five groups and a heat map was drawn (Figure S1A–E). See the Table S3 for detailed data.

Figure S1A shows the heat map analysis of esters in kiwifruit samples. Thirty-seven ester compounds were identified, which contributed to 3.30–93.32% of the total volatile content (TVC), with an average value of 48.31%. In the study of *A. chinensis* [14], a total of 22 esters were detected. Of these, 12 were the same esters as in this study, which may be due to the differences in kiwifruit cultivars and sources. The highest ester content was present in Jingong No. 1 (9575.90 μg/L, 93.32% of TVC), followed by Xuxiang (5264.08 μg/L, 70.48% of TVC), Jinyan (2546.24 μg/L, 48.83% of TVC), Huayou (1756.73 μg/L, 42.66% of TVC), and Qinmei (1463.60 μg/L, 42.45% of TVC). Ethyl acetate, n-propyl acetate, ethyl butanoate, hexyl formate, ethyl hexanoate, and diisobutyl phthalate were the main ester volatiles in kiwifruit. Among them, ethyl acetate and ethyl butanoate were present in all kiwifruit cultivars. Ethyl butanoate had the highest ester content at 7042.01 μg/L (Jinhong No. 1). In the research of Cozzolino et al. [14] on *A. chinensis,* the same conclusion was obtained. Ethyl butanoate was the most abundant substance in ester. Ethyl butanoate, which is fruity and sweet, is found in many other fruits, such as mango [19] and melon [20]. Based on this, ethyl butanoate is one of the esters that has a greater effect on the aroma components of kiwifruit samples and is regarded as one of the core aroma components in kiwifruit.

The lowest ester content was present in Hayward (64.90 μg/L, 3.74% of TVC), and the lowest proportion of esters in TVC was found in Sungold (341.80 μg/L, 3.30% of TVC). The ester concentration in Hort16A was 77.59 μg/L, 7.20% of TVC. Therefore, the content of esters in imported cultivars and introduced cultivars was less than that of new Chinese breeds, and most esters provide the fruit with a complex fruit aroma. This indicates that the aroma chemical compositions of new Chinese breeds are more complicated, which may be caused by multiple crosses between cultivars. Among the esters, there were a total of 11 unique volatiles. They were present in five kiwifruit cultivars, of which Qinmei had a maximum of four unique volatiles, followed by three in Xuxiang, two in Huayou, and one in Jinhong No. 1 and Oriental Red.

Figure S1B is a heat map analysis of alcohols and aldehydes in kiwifruit samples. Seventeen alcohol compounds were identified, which contributed to 0.88–70.73% of the TVC, with an average value of 35.81%. Thirteen aldehydes contributed to 0.86–77.57% of the TVC, with an average value of 39.22%. In alcohols, the main volatiles were ethanol and (E)-2-hexen-1-ol. The main volatile in aldehydes was (E)-2-hexenal, and the secondary volatiles were ethyl aldehyde, hexanal, and decanal. Ethanol had the highest alcohol content (1395.23 μg/L) in Puyu, but its taste was not obvious in kiwifruit samples because of its high threshold (100,000.00 μg/kg) and its ease of escape [21]. The concentration of (E)-2-hexen-1-ol was 1.29–294.54 μg/L, and the concentration was highest in Jinshi. Among aldehydes, (E)-2-hexenal had the highest aldehyde content (6227.87 μg/L) in Sungold, and it was present in all kiwifruit cultivars. Therefore, (E)-2-hexen-1-ol and (E)-2-hexenal were the components among alcohols and aldehydes that had a greater effect on the aroma of kiwifruit, and these were regarded as core aroma components of kiwifruit. In addition, the highest aldehyde concentration and proportion were present in Sungold (8031.25 μg/L, 77.57% of TVC). The main aroma of aldehydes is the taste of green grass and vegetables, which indicates that the grassy aroma of Sungold is more prominent.

Figure S1C shows a heat map analysis of ketones, acids, and alkanes in kiwifruit samples. Sixteen ketone compounds were identified, which contributed to 0.00–5.63% of the TVC, with an average value of 2.82%. Four acids contributed to 0.00–0.89% of the TVC, for which the average value was 0.45%, and twenty alkanes contributed to 0.00–3.89% of the TVC, for which the average value was 1.95%. There were no primary volatiles in ketones, acids, or alkanes. Ketones were not detected in Hongshi No. 2. In Jinhong No. 1 and Sungold, alkanes were not detected. Acids were detected only in Hongshi No. 2, Hongyang, Jinhong 50, Guichang, Jingong No. 7, and Qinmei. Based on this, ketones, acids, and alkanes seem to contribute only marginally to kiwifruit aroma composition. 

Figure S1D shows a heat map analysis of alkenes in kiwifruit samples. Forty alkene compounds were identified, which contributed to 0.18–31.50% of the TVC, with an average value of 15.84%. Alkenes are the category that contains the most volatiles. Among alkenes, there were three primary volatiles, namely, styrene, D-limonene, and 3-isopropylidene-6-methyl-1-cyclohexene. Among them, 3-isopropylidene-6-methyl-1-cyclohexene showed the highest alkene content (724.50 μg/L) in Huayou. The highest alkene content overall was found in Sungold (1276.89 μg/L, 12.33% of TVC), followed by Huayou (1060.51 μg/L, 25.75% of TVC). Most alkenes have a sour taste of lemons or oranges, which indicates that the aroma characteristics of Sungold are more acidic than those of new Chinese breeds.

Figure S1E shows a heat map analysis of aromatic hydrocarbons and their homologs and other components in kiwifruit samples. Sixteen aromatic hydrocarbons and their homologs were identified, which contributed to 0.19–5.34% of the TVC, with an average value of 2.77%. There were two primary volatiles, namely, o-xylene and 3,5-bis(1,1-dimethylethyl)-phenol. The highest aromatic hydrocarbon and homolog contents were present in Jinshi (267.43 μg/L, 5.16% of TVC). The lowest aromatic hydrocarbon and homolog contents were present in Jinfu (8.97 μg/L, 0.29% of TVC). In addition to the above eight categories of volatiles, there were nine other volatiles, but their contents were low. Thus, they were not considered primary components influencing the aroma of kiwifruit.

According to the research of Cozzolino et al. [14] on *A. chinensis*, 72 volatiles were detected, approximately 70% of which were consistent with the results measured in our paper. Meanwhile, the study showed that the volatile profile of all six kiwifruit cultivars was mainly composed of C6 aldehydes and alcohols and the ester ethyl butanoate. This result is consistent with this study. The results in our paper indicate that esters, aldehydes, and alcohols are the main volatiles in kiwifruit. The esters provide fruit sweetness to kiwifruit, while the aldehydes and alcohols provide fresh grassy fragrance. Ethyl butanoate, (E)-2-hexen-1-ol, and (E)-2-hexenal are the core aroma components in kiwifruit. This conclusion is similar to previous research showing that ethyl butanoate, (E)-2-hexen-1-ol, and (E)-2-hexenal have a certain impact on aroma in many kiwifruit cultivars, such as Hayward, Hort16A [13], and Hongyang [9]. In summary, it could be concluded that the volatile components of kiwifruit cultivars are mainly composed of C6 aldehydes, alcohols, and ethyl butanoate. Based on the series comparative analysis of the aroma chemical composition among different cultivars, ethyl butanoate, (E)-2-hexen-1-ol, and (E)-2-hexenal could be considered the core aroma components in kiwifruit, but still need further confirmation using Sensomics in the future.

#### 3.1.4. Classification of Kiwifruit Samples Based on Volatile Components

**Figure 2:**
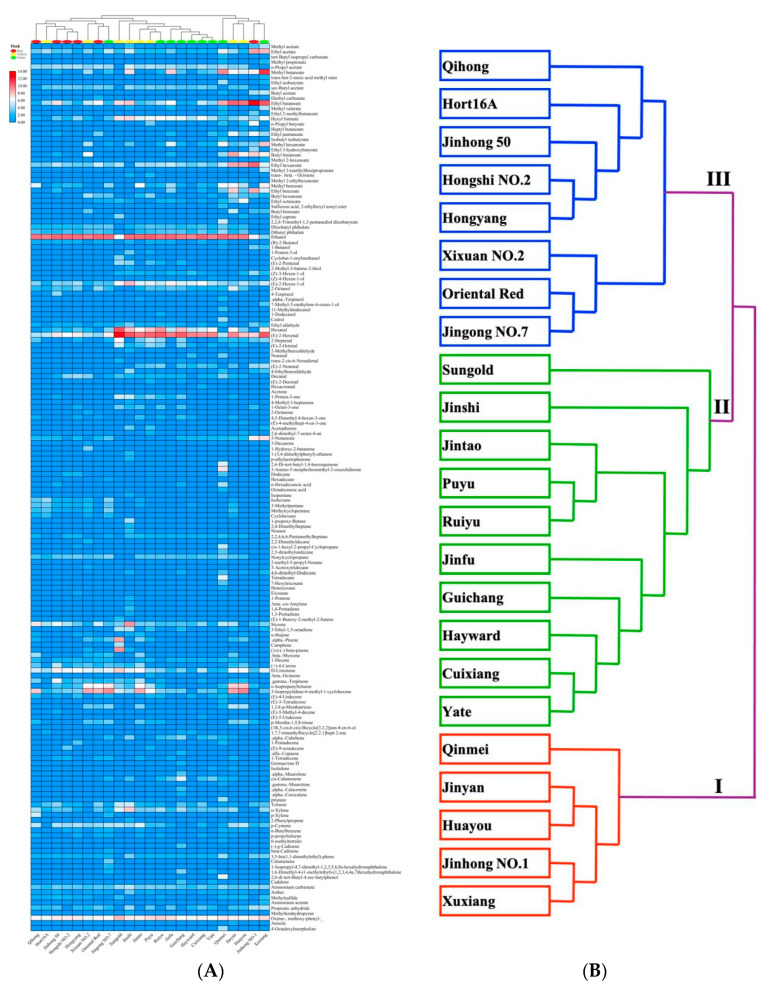
Cluster heat map analysis (**A**) was performed on all volatile components in the kiwifruit samples, and a simplified cluster map (**B**) is presented (I–III: three major categories based on the GC-MS date).

To better understand the relationship of aroma chemical compositions among kiwifruit samples, the kiwifruit samples were further classified by cluster analysis. Figure 2A shows the cluster heat map analysis of kiwifruit samples based on the types and concentrations of volatile components. Figure 2B extracts the clustering part from Figure 2A and draws a simplified diagram to make the results clearer and more intuitive.

Through cluster analysis, 23 kiwifruit samples were preliminarily divided into three major categories. The first category contains five kiwifruits, namely, Xuxiang, Jinhong No. 1, Huayou, Jinyan, and Qinmei. The second category contains 10 kiwifruits, namely, Yate, Cuixiang, Hayward, Guichang, Jinfu, Ruiyu, Puyu, Jintao, Jinshi, and Sungold. There were eight kiwifruits in the third category, namely, Jingong No. 7, Oriental Red, Xixuan No. 2, Hongyang, Hongshi No. 2, Jinhong 50, Hort16A, and Qihong. The second category contains the most kiwifruit cultivars. The imported cultivar Sungold and one of the introduced cultivars, Hayward, belong to the second category, while Hort16A, the other introduced cultivar, belongs to the third category. The results showed that there were no significant differences in aroma chemical compositions between imported and introduced cultivars and new Chinese breeds. In addition, PCA was performed based on GC-MS data (Table S4), and the cumulative variance of the first three principal components was 45.643%, which was less than 80%. Therefore, the results of GC-MS cannot be used to distinguish different kiwifruit cultivars, which is consistent with the clustering results.

### 3.2. Aroma Characteristics of Kiwifruits Characterized by E-Nose

#### 3.2.1. E-Nose Response to Kiwifruit Samples

The aroma of kiwifruit is one of the important characteristics for consumer acceptance and preference for high-quality kiwifruit, and it is also one of the intuitive feelings of consumers when judging the quality of kiwifruit. To more accurately simulate consumers’ perception of the aroma of kiwifruit, based on HS-SPME-GC-MS analysis, E-nose was used to analyze the aroma characteristics of 23 kiwifruit cultivars. Figure S2 shows the E-nose responses of different kiwifruit samples. The x-axis is time, and the y-axis is the relative resistivity, that is, the ratio of sample gas to pure air resistivity (G/G0). Each curve represents the change trend of the corresponding sensor, eventually reaching equilibrium over time, and all varieties of kiwifruit samples reach equilibrium within 50–70 s.

For all kiwifruit samples, S7 (sensitive to many sulfur organic compounds and terpenes), S2 (sensitive to many sulfur organic compounds and terpenes), S6 (sensitive to many sulfur organic compounds and terpenes), and S8 (alcohol, sensitive to aromatic compounds with broad range, similar to No.6) response values increased significantly, while the response values of the remaining sensors hardly changed. After equilibrium, the response values of different varieties of kiwifruit showed significant differences. According to the response values of different sensors after balancing, all varieties of kiwifruit were roughly divided into four categories, which are shown as A–D in Figure S2. In category A kiwifruit, S7 had the highest response value, followed by S6, S2, and S8. In category B kiwifruit, the response value order was S7 > S2 > S6 > S8. In category C kiwifruit, the response value was, from high to low, S2, S7, and S6. In category D kiwifruit, the response values were S6, S7, S8, and S2 from high to low. Among the above four types of kiwifruits, S7 showed a higher response value, while in category C kiwifruits, S8 showed almost no change, and S2 and S7 were almost equal after reaching equilibrium. The response trend curve of the imported cultivar Sungold was category A, and its more prominent characteristic was that it reached equilibrium faster and the fragrance loss was slower. The introduced cultivar Hort16A also belonged to category A. However, overall, there was no significant difference between the two and other category A kiwifruits. The introduced variety Hayward belonged to category B, and there was no significant difference from other category B kiwifruits.

Du et al. [9] obtained the same trend in aroma research of red-fleshed kiwifruit as we did in this experiment. Studies showed that the response values of S7, S2, S6, and S8 increased significantly, while the response values of the remaining sensors hardly changed. However, the response values were different, which may be caused by different instrument settings or samples.

#### 3.2.2. Classification of Kiwifruits Using LDA and Clustering Analysis

**Figure 3:**
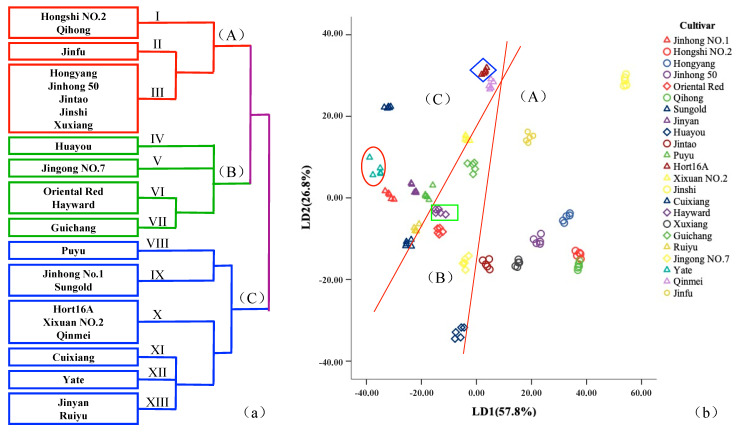
The results of cluster analysis (**a**) and LDA (**b**) (Sungold: red circle; Hayward: green box; Hort16A: blue rhombus) of different varieties of kiwifruit based on E-nose response data. (A–C: three major categories based on the E-nose response).

To further understand the relationship of overall aroma characteristics of different kiwifruit samples, LDA and cluster analysis were performed based on the E-nose response to effectively distinguish and classify kiwifruit samples through aroma characteristics. According to the results of cluster analysis (Figure 3a), samples could be roughly divided into three major categories and 13 minor categories. The first major category was category A, which contained three minor categories and a total of eight kiwifruit cultivars: Hongshi No. 2, Qihong, Jinfu, Hongyang, Jinhong 50, Jintao, Jinshi, and Xuxiang. The second major category was category B, which contained four minor categories. There was a total of five kiwifruit cultivars: Huayou, Jingong No. 7, Oriental Red, Hayward, and Guichang. The third category was category C kiwifruit, which contained six minor categories, and there was a total of 10 kiwifruits: Puyu, Jinhong No. 1, Sungold, Hort16A, Xixuan No. 2, Qinmei, Cuixiang, Yate, Jinyan, and Ruiyu. The third minor category contained the most kiwifruit cultivars, with five kiwifruits. The imported cultivar Sungold was located in the ninth minor category of category C, which was closest to the aroma characteristics of Jinhong No. 1. The introduced cultivar Hort16A belonged to the tenth minor category of category C, which was closer to the aroma characteristics of Xixuan No. 2 and Qinmei. The introduced cultivar Hayward belonged to the sixth minor category of category B, which was closer to the aroma characteristics of Oriental Red.

During the HS-SPME-GC-MS analysis, a cluster analysis was also performed based on the types and concentrations of volatile components (Figure 2) and obtained similar results to those of the E-nose (Figure 3a). A, B, and C were used to represent the three categories of kiwifruit clustered based on E-nose response clustering, and I, II, and III to represent the three categories of kiwifruit clustered based on the GC-MS results. It was found that Hongshi No. 2, Qihong, Hongyang, and Jinhong 50 in category A belonged to category III kiwifruit and were relatively similar, while in category A, Jintao, Jinfu, and Jinshi belonged to category II kiwifruit. Jingong No. 7, Oriental Red, Hayward, and Guichang of category B belonged to category III and category II kiwifruits, and both of them were close. Puyu, Ruiyu, and Sungold belonged to category C and category II kiwifruits. From Figure 2B, it can be seen that although Jinhong No. 1, Jinyan, Qinmei, Yate, and Cuixiang belonged to categories I and II, their aroma characteristics were still close, and all belonged to category C kiwifruit. Similarly, although Xuxiang and Huayou belonged to categories A and B, their aroma characteristics were still close, and both belonged to category I kiwifruit, so they both had a certain degree of similarity. Therefore, after cluster analysis based on the E-nose response and the types and components of volatile components determined by GC-MS, the results showed a certain consistency. 

To determine whether the E-nose could distinguish different varieties of kiwifruit samples, LDA analysis was performed. As shown in Figure 3b, the cumulative variance of the first two-gauge linear discriminant functions (LD) reached 84.60% (greater than 80%), which shows that the E-nose could clearly distinguish different varieties of kiwifruit samples. At the same time, the results of LDA and cluster analysis were basically the same, so the E-nose effectively distinguished different varieties of kiwifruit. Combining LDA and cluster analysis, it was found that the overall aroma characteristics of the imported cultivar Sungold and the two introduced cultivars Hayward and Hort16A were not significantly different from the overall aroma characteristics of the new Chinese breeds.

#### 3.2.3. Analysis of the Differences in E-Nose Responses Based on Volatile Components

**Figure 4:**
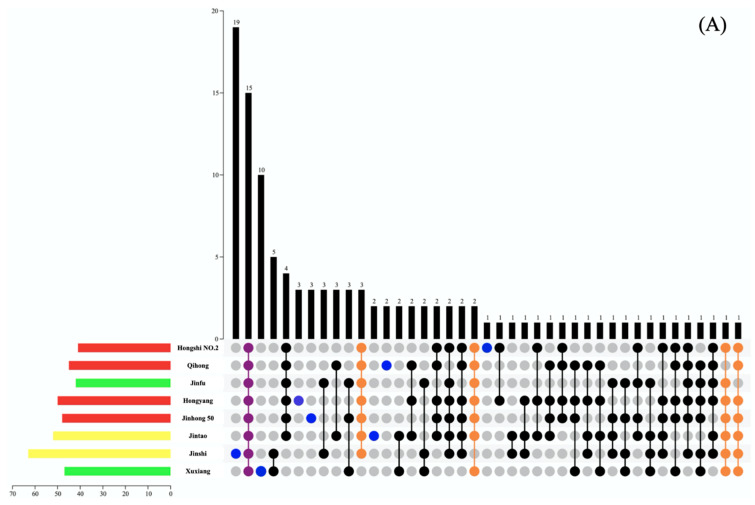

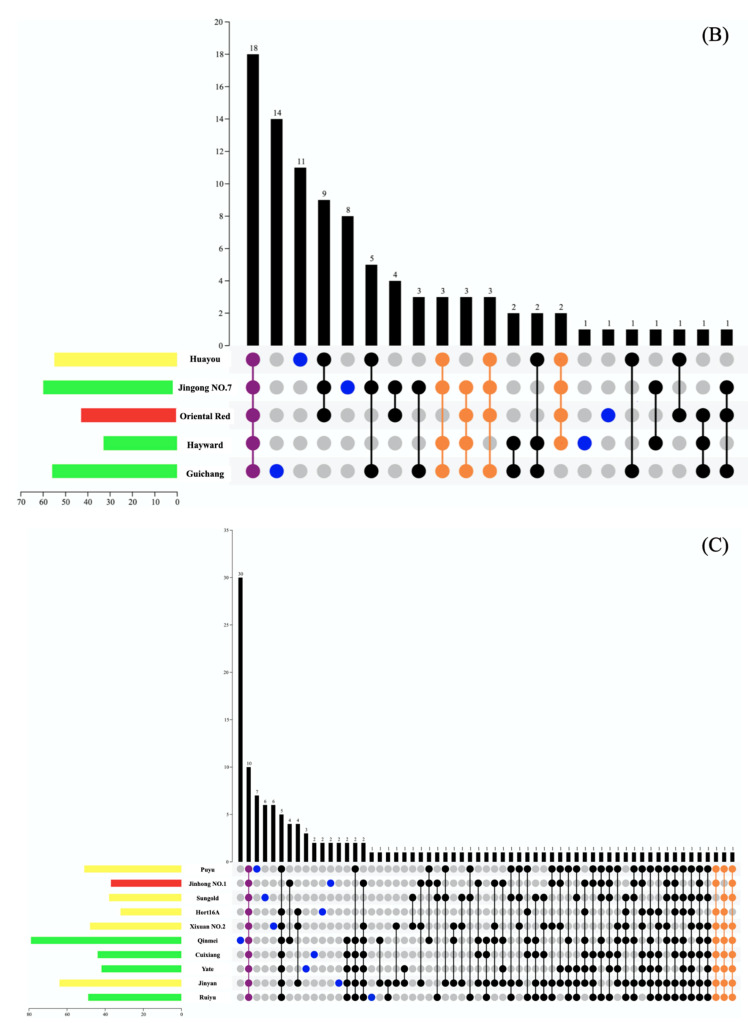
Venn diagrams for the analysis of volatile components in kiwifruit samples of different types (three major categories (**A**–**C**) based on the E-nose response). The length of the histogram on the left represents the number of volatile components in different kiwifruit cultivars, and the color represents the color of the kiwifruit flesh, namely, green-, yellow-, and red-flesh kiwifruit. The dot chart below represents the number of common volatile components in different kiwifruit cultivars.

In the previous analysis, cluster analysis and LDA were performed (Figure 3) on kiwifruit samples based on the E-nose response, and the kiwifruit samples were initially divided into three major categories and 13 minor categories. The classification results were similar to those of GC-MS (Figure 2). To further understand the relationship between GC-MS and E-nose analysis, the method of drawing Venn diagrams was used to analyze the differences in the E-nose responses based on volatile components (see Figure 4 for details).

In this study, Venn diagrams were used to analyze the types of volatile components in three categories of kiwifruit samples, namely, A, B, and C, which were divided based on E-nose response. Taken together, there were nine volatile components common to category A, B, and C kiwifruits. Among the common components of category A kiwifruit, there were five volatiles that were unique to category B and C kiwifruits, namely, ethyl aldehyde, hexyl formate, o-xylene, p-cymene, and 3-isopropylide-6-methyl-1-cyclohexene. Among the common components of category B kiwifruit, there were eight volatiles that were unique to category A and C kiwifruits, namely, n-propyl acetate, sec-butyl acetate, 2-octanol, 1-decene, 3-nonnanone, nonylcyclopropane, diisobutyl phthalate, and dibutyl phthalate. The common components of category C kiwifruit were found in category A or B, which indicates that the unique volatiles in category A and B kiwifruits may have a greater impact on the overall aroma characteristics of kiwifruit, resulting in certain differences in the response of the E-nose among the three categories of kiwifruit.

In summary, the similarity of the volatile components in category B kiwifruit was the highest, while the similarity in category C kiwifruit was the lowest. There were many cultivars of kiwifruit samples among category C kiwifruit, which caused the similarity of volatile components to decrease. Among all kiwifruit samples, Qinmei had the most unique volatiles (30), followed by Jinshi (20). Jinfu did not contain unique volatile components. This could increase understanding of the relationship between GC-MS and E-nose analysis.

### 3.3. Relationship between Aroma Chemical Compositions of Kiwifruit Flesh Color and Species

Based on the analysis of the aroma chemical compositions of different cultivars of kiwifruit, the relationship between the aroma characteristics of kiwifruit samples and their flesh colors or varieties were further analyzed. A total of 23 cultivars of kiwifruit were used, including 6 red-fleshed kiwifruits (3 yellow- and red-fleshed (middle part) kiwifruits and 3 green- and red-fleshed (middle part) kiwifruits), 8 yellow-fleshed kiwifruits, and 9 green-fleshed kiwifruits. There were 15 *A. chinensis* fruits and 8 *A. deliciosa* fruits.

**Figure 5:**
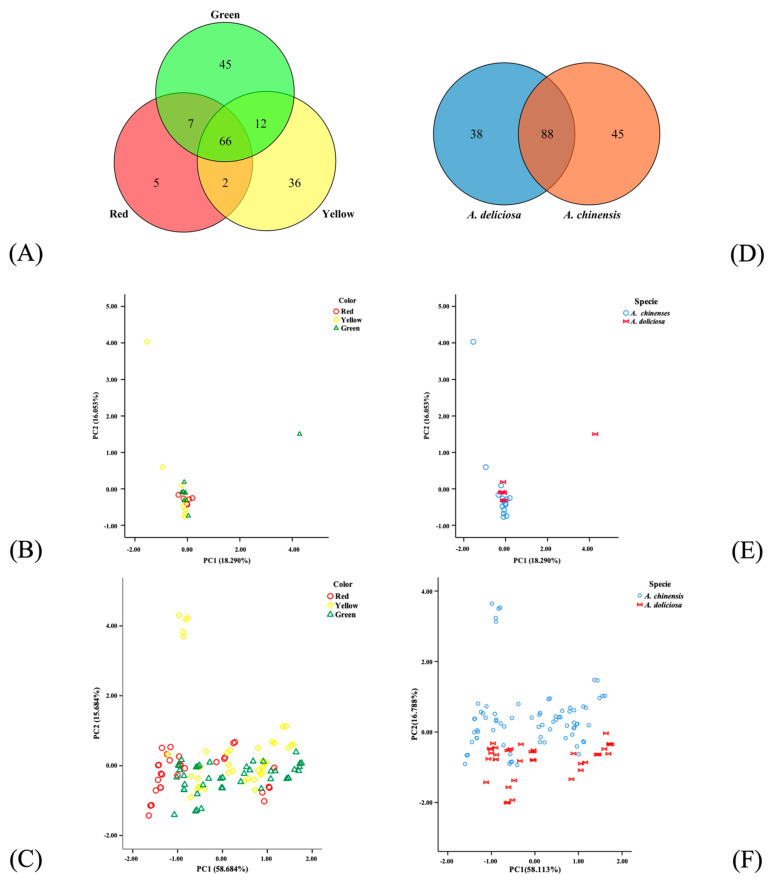
Venn diagrams for analysis of volatile components in kiwifruit samples with different flesh colors (**A**) and varieties (**D**). The results of PCA of kiwifruit with different flesh colors based on GC-MS data (**B**) and E-nose response data (**C**). The results of PCA of kiwifruit of different varieties based on GC-MS data (**E**) and E-nose response data (**F**).

Based on the kinds of volatile components, Venn diagrams was used to analyze kiwifruit samples with different flesh colors (Figure 5A) and different species (Figure 5D). Red-, yellow-, and green-fleshed kiwifruits contained 80, 116, and 130 volatile components, respectively. Among them, 66 volatile components were common. Green- and yellow-fleshed kiwifruits shared 12 volatile components, green- and red-fleshed fruits shared 7 volatile components, and red- and yellow-fleshed kiwifruits shared only 2 volatile components. Based on these species, 88 volatile components were found commonly in *A. chinensis* and *A. deliciosa*, 45 volatile components were unique to *A. chinensis,* and 38 volatile components were unique to *A. deliciosa*.

According to the E-nose responses, 23 kiwifruit samples in this study were analyzed by PCA based on flesh color and species. Figure 5C shows the PCA of kiwifruit with different flesh colors based on the E-nose response. Principal component analysis showed that 74.37% of the variance was explained by the first two components of the PCA. The sampling points of kiwifruit samples with different flesh colors were mostly mixed together and could not be distinguished. The most prominent cultivar was the yellow-fleshed cultivar Jinshi, which was significantly different from other cultivars of kiwifruit. However, the overall results show that there were no significant differences in the aroma characteristics of kiwifruits with different flesh colors. Figure 5F shows the PCA of kiwifruit of different species based on the E-nose response. When the samples overlapped or were close to each other, they had similar flavors. The first two principal components accounted for 74.90% of the total variance, and some sampling points overlapped. This indicates that the aroma characteristics of kiwifruits with different species were different in general, but some samples were similar. Therefore, the E-nose could not effectively distinguish and identify kiwifruit samples of different flesh colors, although it could distinguish kiwifruit samples of different species to a certain extent, and it cannot be used for identification. This may be due to the current kiwifruit cultivars undergoing multiple crosses between species during the cultivation process, resulting in smaller differences between kiwifruit cultivars. Therefore, aroma characteristics have little association with flesh color and species. However, the PCA results based on GC-MS data show that neither the flesh color nor the species can be distinguished (Figure 5B,E).

On the whole, kiwifruit samples of different flesh colors cannot be effectively distinguished by their aroma chemical compositions with the approach presented here. Based on the types of volatile components, green-fleshed kiwifruit has the most volatile components and the most complex aroma. The types of volatile components of yellow- and green-fleshed kiwifruit are more similar. Kiwifruit samples of different species can be distinguished to some extent by E-nose.

Zhang et al. [11] analyzed the GC-MS results by PCA and found that the volatile compounds of *A. chinensis* and *A. deliciosa* were similar, which is basically consistent with the conclusions drawn in our study. The reason may be that the two species are closely related, and *A. deliciosa* is a variant of *A. chinensis* [22].

## 4. Conclusions

In summary, the aroma chemical compositions of 23 kiwifruit samples were analyzed through GC-MS and E-nose. The results indicated that 2-octanone and 3-octanone could be used as dual internal standard substances in HS-SPME-GC-MS detection and have the highest general applicability, with the smallest error (0.22–4.89% RSD). A total of 172 effective aroma components were detected, which were divided into 9 categories according to functional groups, including 37 esters, 17 alcohols, 13 aldehydes, 16 ketones, 4 acids, 20 alkanes, 40 alkenes, 16 aromatic hydrocarbons and their homologues, and 9 other components. Among them, there were 16 main volatiles, 21 secondary volatiles, and 74 unique volatiles in kiwifruit. Esters, alcohols, and aldehydes had a greater effect on the aroma characteristics of kiwifruit. Ethyl butanoate, (E)-2-hexen-1-ol, and (E)-2-hexenal are core aroma components in kiwifruit, but still need further confirmation using Sensomics in the future. Moreover, the E-nose can effectively distinguish different cultivars of kiwifruit. Clustering based on the results of GC-MS and E-nose indicated a certain degree of similarity. However, kiwifruit cultivars with different flesh colors cannot be effectively distinguished by their aroma chemical compositions, while different species of kiwifruit (*A. chinensis* and *A. deliciosa*) can be distinguished to some extent, but the effect was not satisfactory. The results show that the aroma chemical compositions of new Chinese breeds are more complicated, while for Sungold, the aroma dissipation rate is slower, and the aroma retention time is longer. These results provide a certain theoretical basis for the future breeding, planting, and marketing of kiwifruit.

## Figures and Tables

**Table 1:** The selection of internal standards.

No	Cultivar	Internal Quantitative Standard	Internal Verification Standards	%RSD ^a^
1	Jinhong NO.1	2-Octanone	(S)-(+)-2-Octanol	2.84
2	Hongshi NO.2	2-Octanone	3-Octanone	3.38
3	Hongyang	2-Octanone	3-Octanone	3.13
4	Jinhong 50	2-Octanone	3-Octanone	2.19
5	Oriental Red	2-Octanone	3-Octanone	4.29
6	Qihong	2-Octanone	3-Octanone	1.18
7	Sungold	2-Octanone	(S)-(+)-2-Octanol	8.91
8	Jinyan	2-Octanone	3-Octanone	2.34
9	Huayou	2-Octanone	3-Octanone	2.50
10	Jintao	2-Octanone	3-Octanone	3.01
11	Puyu	2-Octanone	3-Octanone	1.53
12	Hort16A	2-Octanone	3-Octanone	0.22
13	Xixuan NO.2	2-Nonanone	3-Nonanone	10.81
14	Jinshi	3-Octanone	3-Octanol	15.41
15	Cuixiang	2-Octanone	3-Octanone	2.83
16	Hayward	2-Octanone	3-Octanone	1.06
17	Xuxiang	2-Octanone	(S)-(+)-2-Octanol	2.29
18	Guichang	2-Octanone	3-Octanone	4.89
19	Ruiyu	2-Octanone	3-Octanone	1.17
20	Jingong NO.7	2-Octanone	3-Octanone	3.77

^a^ The relative standard deviation between the internal quantification standard and the internal verification standard.

**Table 2:** The volatile components in kiwifruit and their basic parameters and concentration range.

NO.	Compounds	RI ^a^	CAS ^b^	Concentration Range (μg/L)	Cultivars ^c^
***Esters***					
1	Methyl acetate	487	79-20-9	0.00–25.56	3
2	Ethyl acetate	586	141-78-6	1.42–430.85	23 ^S,A,H^
3	*tert*-Butyl isopropyl carbonat	911	-	0.00–3.42	1
4	Methyl propionate	586	554-12-1	0.00–4.14	2
5	n-Propyl acetate	686	109-60-4	0.00–43.27	20 ^A,H^
6	Methyl butanoate	686	623-42-7	0.00–2675.60	14
7	*trans*-but-2-enoic acid methyl ester	694	623-43-8	0.00–2.53	1
8	Ethyl isobutyrate	721	97-62-1	0.00–20.84	2
9	*sec*-Butyl acetate	721	105-46-4	0.00–15.72	18 ^A,H^
10	Butyl acetate	785	123-86-4	0.00–29.77	3
11	Diethyl carbonate	761	105-58-8	0.00–3.54	1
12	Ethyl butanoate	785	105-54-4	1.56–7042.01	23 ^S,A,H^
13	Methyl valerate	785	624-24-8	0.00–23.31	4
14	Ethyl 2-methylbutanoate	820	7452-79-1	0.00–9.62	2
15	Hexyl formate	981	629-33-4	0.00–167.99	21 ^S,H^
16	n-Propyl butyrate	884	105-66-8	0.00–85.50	4
17	Heptyl butanoate	1282	5870-93-9	0.00–1.36	1
18	Ethyl pentanoate	884	539-82-2	0.00–66.12	9
19	Isobutyl isobutyrate	856	97-85-8	0.00–3.86	1
20	Methyl hexanoate	884	106-70-7	0.00–226.78	13
21	Ethyl 3-hydroxybutyrate	947	5405-41-4	0.00–16.37	1
22	Butyl butanoate	984	109-21-7	0.00–409.61	8
23	Methyl 2-hexenoate	892	2396-77-2	0.00–2.10	1
24	Ethyl hexanoate	984	123-66-0	0.00–1223.14	22 ^S,A,H^
25	Methyl 3-(methylthio)propionate	936	13532-18-8	0.00–25.71	1
26	*trans*-β-Ocimene	976	3779-61-1	0.00–3.81	2
27	Methyl 2-ethylhexanoate	1019	816-19-3	0.00–1.32	1
28	Methyl benzoate	1060	93-58-3	0.00–104.95	16
29	Ethyl benzoate	1160	93-89-0	0.00–349.13	8
30	Butyl hexanoate	1183	626-82-4	0.00–22.84	10
31	Ethyl octanoate	1183	106-32-1	0.00–64.28	11 ^S^
32	Sulfurous acid, 2-ethylhexyl nonyl ester	2270	-	0.00–4.72	1
33	Butyl benzoate	1359	136-60-7	0.00–32.36	4
34	Ethyl caprate	1381	110-38-3	0.00–14.15	2 ^S^
35	2,2,4-Trimethyl-1,3-pentanediol diisobutyrate	1605	6846-50-0	0.00–36.52	1
36	Diisobutyl phthalate	1908	84-69-5	0.00–8.78	20 ^A,H^
37	Dibutyl phthalate	2037	84-74-2	0.00–15.77	19 ^A,H^
***Alcohols***					
38	Ethanol	463	64-17-5	113.69–1395.23	23 ^S,A,H^
39	(R)-2-Butanol	581	14898-79-4	0.00–2.56	13 ^A^
40	1-Butanol	662	71-36-3	0.00–10.52	3
41	1-Penten-3-ol	671	616-25-1	0.00–9.39	2
42	Cyclobut-1-enylmethanol	794	89182-08-1	0.00–12.89	1^S^
43	(E)-2-Pentenal	715	1576-87-0	0.00–19.19	5^S^
44	2-Methyl-3-butene-2-thiol	807	5287-45-6	0.00–1.77	1
45	(Z)-3-Hexen-1-ol	868	928-96-1	3.11–17.50	13 ^S,H^
46	(Z)-4-Hexen-1-ol	868	928-91-6	0.00–0.63	1
47	(E)-2-Hexen-1-ol	868	928-95-0	11.53–294.54	23 ^S,A,H^
48	2-Octanol	979	123-96-6	0.00–45.94	18 ^A,H^
49	4-Terpineol	1137	562-74-3	0.00–15.85	1
50	α-Terpineol	1143	98-55-5	0.00–3.95	2
51	7-Methyl-3-methylene-6-octen-1-ol	1228	13066-51-8	0.00–6.47	1
52	11-Methyldodecanol	1492	85763-57-1	0.00–5.44	1
53	1-Dodecanol	1457	112-53-8	0.00–1.67	1
54	Cedrol	1543	77-53-2	0.00–18.44	1
***Aldehydes***					
55	Ethyl aldehyde	408	75-07-0	0.00–10.07	17 ^S^
56	Hexanal	806	66-25-1	0.00–1671.57	19 ^S,A,H^
57	(E)-2-Hexenal	814	6728-26-3	15.89–6227.87	23 ^S,A,H^
58	2-Heptenal	913	57266-86-1	0.00–79.07	13 ^S^
59	(E)-2-Octenal	1013	2548-87-0	0.00–39.58	10 ^S,H^
60	2-Methylbenzaldehyde	1095	529-20-4	0.00–2.30	1
61	Nonanal	1104	124-19-6	0.00–9.77	2 ^H^
62	*trans*-2-*cis*-6-Nonadienal	1120	557-48-2	0.00–2.90	2 ^S^
63	(E)-2-Nonenal	1112	18829-56-6	0.00–22.92	10 ^H^
64	4-Ethylbenzaldehyde	1195	4748-78-1	0.00–16.44	4
65	Decanal	1204	112-31-2	0.00–18.70	17 ^S^
66	(E)-2-Decenal	1212	3913-81-3	0.00–2.91	2
67	Hexacosanal	-	26627-85-0	0.00–1.85	1
***Ketones***					
68	Acetone	455	67-64-1	0.00–0.20	1^A^
69	1-Penten-3-one	644	1629-58-9	0.00–38.43	9 ^S^
70	4-Methyl-3-heptanone	888	6137-11-7	0.00–1.77	1
71	1-Octen-3-one	943	4312-99-6	0.00–9.21	7 ^S^
72	2-Octanone	952	111-13-7	0.00–1.79	1
73	4,5-Dimethyl-4-hexen-3-one	915	17325-90-5	0.00–5.31	8 ^H^
74	(E)-4-methylhept-4-en-3-one	938	22319-31-9	0.00–1.00	1
75	Acetophenon	1029	98-86-2	0.00–8.91	3
76	2,6-dimethyl-7-octen-4-on	-	1879-00-1	0.00–2.10	2
77	3-Nonanone	1052	925-78-0	0.00–145.93	15 ^A,H^
78	3-Decanone	1151	928-80-3	0.00–0.83	1
79	1-Hydroxy-2-butanone	798	5077-67-8	0.00–11.75	1
80	1-(3,4-dimethylphenyl)-ethanon	1255	3637-01-2	0.00–4.52	1
81	p-ethylacetophenone	1242	937-30-4	0.00–3.98	1
82	2,6-Di-tert-butyl-1,4-benzoquinone	1633	719-22-2	0.00–49.68	1
83	3-Amino-5-morpholinomethyl-2-oxazolidinone	1844	43056-63-9	0.00–140.65	1
***Acids***					
84	Dodecane	1214	112-40-3	0.00–6.26	3
85	Hexadecane	1612	544-56-3	0.00–0.90	1
86	n-Hexadecanoic acid	1968	57-10-3	0.00–21.51	3
87	Octadecanoic acid	2167	57-11-4	0.00–3.00	1
***Alkanes***					
88	Isopentane	454	78-78-4	0.00–1.98	1
89	Isohexane	554	107-83-5	0.00–17.16	9 ^A^
90	3-Methylpentane	554	96-14-0	0.00–8.89	10 ^A,H^
91	Methylcyclopentane	661	96-37-7	0.00–10.79	8 ^A^
92	Cyclohexane	719	110-82-7	0.00–5.14	5 ^A^
93	1-propoxy-Butane	793	3073-92-5	0.00–21.23	1
94	2,4-Dimethylheptane	788	2213-23-2	0.00–3.43	1
95	Nonane	916	111-84-2	0.00–4.42	1
96	2,2,4,6,6-Pentamethylheptane	981	13475-82-6	0.00–4.78	16 ^H^
97	2,2-Dimethyldecane	1130	17302-37-3	0.00–3.65	1
98	*cis*-1-hexyl-2-propyl-Cyclopropane	1178	74630-58-3	0.00–31.78	2
99	2,5-dimethylundecane	1185	17301-22-3	0.00–6.90	1
100	Nonylcyclopropane	1216	74663-85-7	0.00–13.89	19 ^A,H^
101	2-methyl-5-propyl-Nonane	1185	31081-17-1	0.00–0.84	1
102	3-Acetoxytridecane	1615	-	0.00–1.37	1
103	4,6-dimethyl-Dodecane	1285	61141-72-8	0.00–5.75	5
104	Tetradecane	1413	629-59-4	0.00–53.06	1
105	7-Hexyleicosane	2542	55333-99-8	0.00–7.23	1
106	Heneicosane	2109	629-94-7	0.00–1.71	1
107	Eicosane	-	112-95-8	0.00–1.27	1
***Alkenes***					
108	1-Pentene	508	109-67-1	0.00–2.77	1
109	β-*cis*-Amylene	516	627-20-3	0.00–1.49	1
110	1,4-Pentadiene	498	591-93-5	0.00–5.07	1
111	1,3-Pentadiene	516	504-60-9	0.00–1.93	1
112	(E)-1-Butoxy-2-methyl-2-butene	977	-	0.00–4.57	1 ^S^
113	Styrene	883	100-42-5	0.00–521.27	22 ^S,A,H^
114	3-Ethyl-1,5-octadiene	949	-	0.00–25.25	6 ^S^
115	α-thujene	902	2867-05-2	0.00–3.96	6
116	α-Pinene	948	80-56-8	0.00–406.12	9 ^S^
117	Camphene	943	79-92-5	0.00–22.85	1 ^S^
118	(1s)-(-)-beta-pinene	943	18172-67-3	0.00–543.45	3 ^S^
119	β-Myrcene	958	123-35-3	0.00–18.67	6
120	1-Decene	1005	872-05-9	0.00–4.12	17 ^H^
121	(+)-4-Carene	919	29050-33-7	0.00–27.72	11 ^S^
122	D-Limonene	1018	5989-27-5	1.55–201.63	23 ^S,A,H^
123	β-Ocimene	976	3779-61-1	0.00–2.03	1
124	γ-Terpinene	998	99-85-4	0.00–58.33	14^S^
125	o-Isopropenyltoluene	1073	7399-49-7	0.00–209.16	15 ^S^
126	3-Isopropylidene-6-methyl-1-cyclohexene	1023	586-63-0	0.00–724.50	21 ^S,A^
127	(E)-4-Undecene	1123	693-62-9	0.00–5.05	13 ^H^
128	(E)-3-Tetradecene	1421	41446-68-8	0.00–2.92	1
129	1,3,8-p-Menthatriene	1029	18368-95-1	0.00–28.51	7
130	(E)-5-Methyl-4-decene	1100	62338-51-6	0.00–1.63	1
131	(E)-5-Undecene	-	764-97-6	0.00–1.06	1
132	*p*-Mentha-1,5,8-triene	-	21195-59-5	0.00–13.70	10
133	(1R,5-cis,6-cis)-Bicyclo[3.2.2]non-8-en-6-ol	1152	-	0.00–0.66	1
134	1,7,7- trimethylbicyclo [2.2.1] hept-2-ene	932	464-17-5	0.00–2.06	3 ^A,H^
135	α-Cubebene	1344	17699-14-8	0.00–15.01	9
136	1-Pentadecene	1502	13360-61-7	0.00–6.81	2
137	(E)-9-octadecene	1818	7206-25-9	0.00–7.96	2
138	α-Copaene	1221	-	0.00–3.62	1
139	1-Tetradecene	1403	1120-36-1	0.00–2.70	7
140	Germacrene D	1515	23986-74-5	0.00–2.68	1
141	Isoledene	-	95910-36-4	0.00–1.01	2
142	α-Muurolene	1440	10208-80-7	0.00–6.65	1
143	*cis*-Calamenene	-	72937-55-4	0.00–52.90	9
144	γ-Muurolene	1435	39029-41-9	0.00–1.48	1
145	α-Calacorene	1547	21391-99-1	0.00–5.33	1
146	α-Corocalene	-	20129-39-9	0.00–2.84	1
147	pristane	1653	1921-70-6	0.00–20.11	1
***Aromatic hydrocarbons and their homologues***					
148	Toluene	794	108-88-3	0.00–38.54	19 ^S,A,H^
149	o-Xylene	907	95-47-6	0.00–196.84	20 ^S,A,H^
150	*p*-Xylene	907	106-42-3	0.00–39.14	2
151	2-Phenylpropene	928	98-83-9	0.00–2.64	1
152	*p*-Cymene	1042	99-87-6	0.00–47.77	19 ^S^
153	n-Butylbenzene	1092	104-51-8	0.00–2.16	8
154	*p*-propyltoluene	1106	1074-55-1	0.00–2.11	9
155	6-methyltetralin	1280	1680-51-9	0.00–1.42	1
156	(-)-g-Cadinene	1435	39029-41-9	0.00–6.51	1
157	β-Cadinene	1440	523-47-7	0.00–1.95	1
158	3,5-bis(1,1-dimethylethyl)-phenol	1555	1138-52-9	0.00–19.68	22 ^A,H^
159	Calamenene	1537	483-77-2	0.00–10.05	1
160	1-Isopropyl-4,7-dimethyl-1,2,3,5,6,8a-hexahydronaphthalene	-	16729-01-4	0.00–4.17	1
161	1,6-Dimethyl-4-(1-methylethyl) -(1,2,3,4,4a,7) hexahydronaphthalene	1902	16728-99-7	0.00–12.63	3
162	2,6-di-tert-Butyl-4-sec-butylphenol	1902	17540-75-9	0.00–59.02	1
163	Cadalene	1706	483-78-3	0.00–6.07	2
***Others***					
164	Ammonium carbamate	-	1111-78-0	4.42–28.61	23 ^S,A,H^
165	Aether	495	60-29-7	0.00–9.33	19 ^A^
166	Methylsulfide	471	75-18-3	0.00–4.97	12 ^S,A^
167	Ammonium acetate	630	631-61-8	0.00–3.77	1
168	Propionic anhydride	921	123-62-6	0.00–24.21	17 ^A,H^
169	Methylterahydropyran	770	10141-72-7	0.00–2.23	1
170	methoxy-phenyl-oxime	1301	-	53.75–291.96	23 ^S,A,H^
171	Anisole	870	100-66-3	0.00–1.80	1
172	4-Octadecylmorpholine	2511	16528-77-1	0.00–20.09	1

^a^ Retention index of compounds on DB-1MS chromatographic column columns. ^b^ CAS, Chemical Abstracts Service Registry Number of the odorant. ^c^ Number of kiwifruit cultivars with volatile compounds. ^S^ Volatile components contained in Sungold. ^A^ Volatile components contained in Hort16A. ^H^ Volatile components contained in Hayward.

## Supplementary Materials

The following are available online at https://www.mdpi.com/article/10.3390/foods10071645/s1, Figure S1: Quantitative analysis of esters (A), alcohols and aldehydes (B), ketones, acids and alkanes (C), alkenes (D) and aromatic hydrocarbons and their homologs and other volatile components (E) in kiwifruit samples. The concentration of volatile components gradually increased from blue to red in units of μg/L, Figure S2. Kiwifruits samples were divided into 4 categories based on electronic nose response (A)–(D). Electronic nose sensor (E). Table S1: Cultivars, species, flesh color, region and retail price of kiwifruits, Table S2. The determination of internal standards, Table S3. The concentration of volatile components in different cultivars of kiwifruit, Table S4. Total variance explained.

## References

[1] Ma T.T., Lan T., Geng T.H., Ju Y.L., Cheng G., Que Z.L., Gao G.T., Fang Y.L., Sun X.Y. (2019). Nutritional properties and biological activities of kiwifruit (*Actinidia*) and kiwifruit products under simulated gastrointestinal in vitro digestion. Food Nutr. Res..

[2] Crowhurst R.N., Gleave A.P., MacRae E.A., Ampomah-Dwamena C., Atkinson R.G., Beuning L.L., Bulley S.M., Chagne D., Marsh K.B., Matich A.J. (2008). Analysis of expressed sequence tags from *Actinidia*: Applications of a cross species EST database for gene discovery in the areas of flavor, health, color and pipening. BMC Genom..

[3] Garcia C.V., Stevenson R.J., Atkinson R.G., Winz R.A., Quek S.Y. (2013). Changes in the bound aroma profiles of ‘Hayward’ and ‘Hort16A’ kiwifruit (*Actinidia* spp.) during ripening and GC-olfactometry analysis. Food Chem..

[4] Ma T.T., Sun X.Y., Zhao J.M., You Y.L., Lei Y.S., Gao G.T., Zhan J.C. (2017). Nutrient compositions and antioxidant capacity of kiwifruit (*Actinidia*) and their relationship with flesh color and commercial value. Food Chem..

[5] Henare S.J., Rutherfurd S.M., Drummond L.N., Borges V., Boland M.J., Moughan P.J. (2012). Digestible nutrients and available (ATP) energy contents of two varieties of kiwifruit (*Actinidia deliciosa* and *Actinidia chinensis*). Food Chem..

[6] Sauvageau J., Hinkley S.F., Carnachan S.M., Sims I.M. (2010). Characterisation of polysaccharides from gold kiwifruit (*Actinidia chinensis* Planch. ‘Hort16A’). Carbohydr. Polym..

[7] Sivakumaran S., Huffman L., Sivakumaran S., Drummon L. (2018). The nutritional composition of Zespri^®^ SunGold Kiwifruit and Zespri^®^ Sweet Green Kiwifruit. Food Chem..

[8] Cremon C., Pagano I., Marcellini M.M., Barbaro M.R., Gearry R., Fukudo S., Drummond L., Ansell J., Mauloni P., Capelli E. (2017). The effect of Zespri green kiwifruit on digestive and gut health functions. Neueogastroent. Motil..

[9] Du D., Xu M., Wang J., Gu S., Zhu L., Hong X. (2019). Tracing internal quality and aroma of a red-fleshed kiwifruit during ripening by means of GC-MS and E-nose. RSC Adv..

[10] Monro J.A., Paturi G., Mishra S. (2017). Effects of kiwifruit and mixed dietary fibre on faecal properties and microbiota in rats: A dose-response analysis. Int. J. Food Sci. Technol..

[11] Zhang C.Y., Zhang Q., Zhong C.H., Guo M.Q. (2019). Volatile fingerprints and biomarkers of three representative kiwifruit cultivars obtained by headspace solid-phase microextraction gas chromatography mass spectrometry and chemometrics. Food Chem..

[12] Lim S., Lee J.G., Lee E.J. (2017). Comparison of fruit quality and GC-MS-based metabolite profiling of kiwifruit ‘Jecy green’: Natural and exogenous ethylene-nduced ripening. Food Chem..

[13] Garcia C.V., Quek S.Y., Stevenson R.J., Winz R.A. (2012). Kiwifruit flavour: A review. Trends Food Sci. Technol..

[14] Cozzolino R., Giulio B.D., Petriccione M., Martignetti A., Malorni L., Zampella L., Laurino C., Pellicano M.P. (2020). Comparative analysis of volatile metabolites, quality and sensory attributes of *Actinidia chinensis* fruit. Food Chem..

[15] Ma T.T., Wang J.Q., Wang H.L., Lan T., Liu H.R., Gao T., Yang Y.F., Zhou Y., Ge Q., Fang Y.L. (2020). Is overnight fresh juice drinkable? The shelf life prediction of non-industrial fresh watermelon juice based on the nutritional quality, microbial safety quality, and sensory quality. Food Nutr. Res..

[16] Ma T.T., Wang J.Q., Wang L.K., Yang Y.H., Yang W.Y., Wang H.L., Lan T., Zhang Q.W., Sun X.Y. (2020). Ultrasound-combined sterilization technology: An effective sterilization technique ensuring the microbial safety of grape juice and significantly improving its quality. Foods.

[17] Liu Q., Zhao N., Zhou D.D., Sun Y., Sun K., Pan L.Q., Kang T. (2018). Discrimination and growth tracking of fungi contamination in peaches using electronic nose. Food Chem..

[18] Alexis M.V., Katariina K.M., Saila K., Heikki K., Baoru Y. (2018). Profiles of volatile compounds in blackcurrant (*Ribes nigrum*) cultivars with a special focus on the influence of growth latitude and weather conditions. J. Agric. Food Chem..

[19] Xiao Z.B., Xiang P., Zhu J.C., Zhu Q., Niu Y.W. (2019). Evaluation of the perceptual interaction among sulfur compounds in mango by feller’s additive model, odor activity value, and vector model. J. Agric. Food Chem..

[20] Shi J.D., Wu H.B., Xiong M., Chen Y.J., Chen J.H., Zhou B., Wang H., Li L.L., Fu X.F., Bie Z.L. (2020). Comparative analysis of volatile compounds in thirty nine melon cultivars by headspace solid-phase microextraction and gas chromatography-mass spectrometry. Food Chem..

[21] van Gemert L.J. (2011). Odour Thresholds. Compilations of Odour Threshold Values in Air, Water and Other Media.

[22] Huang H.W., Li Z.Z., Li J.Q., Kubisiak T.L., Testolin R. (2002). Phylogenetic relationships in *Actinidia* as revealed by RAPD analysis. J. Am. Soc. Hortic. Sci..

