# Supplementary material for: Analysis of the Aroma Chemical Composition of Commonly Planted Kiwifruit Cultivars in China
Source: Foods. 2021 Jul 16;10(7):1645. doi: 10.3390/foods10071645 (PMC8306980; doi:10.3390/foods10071645)
Supplement: Supplementary file 1 [file foods-10-01645-s001.zip › foods-1279381-supplementary.pdf]

Supplementary Materials:

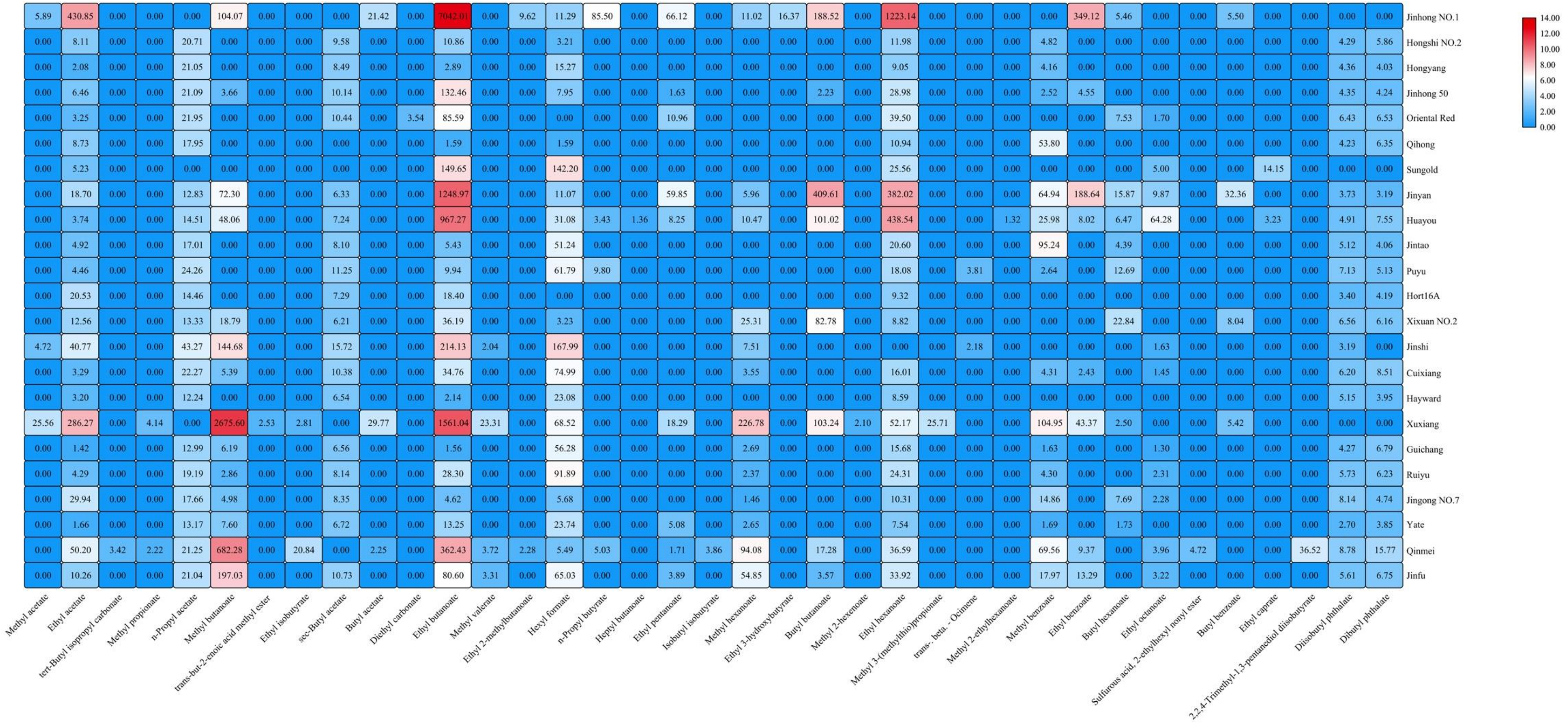

(A)

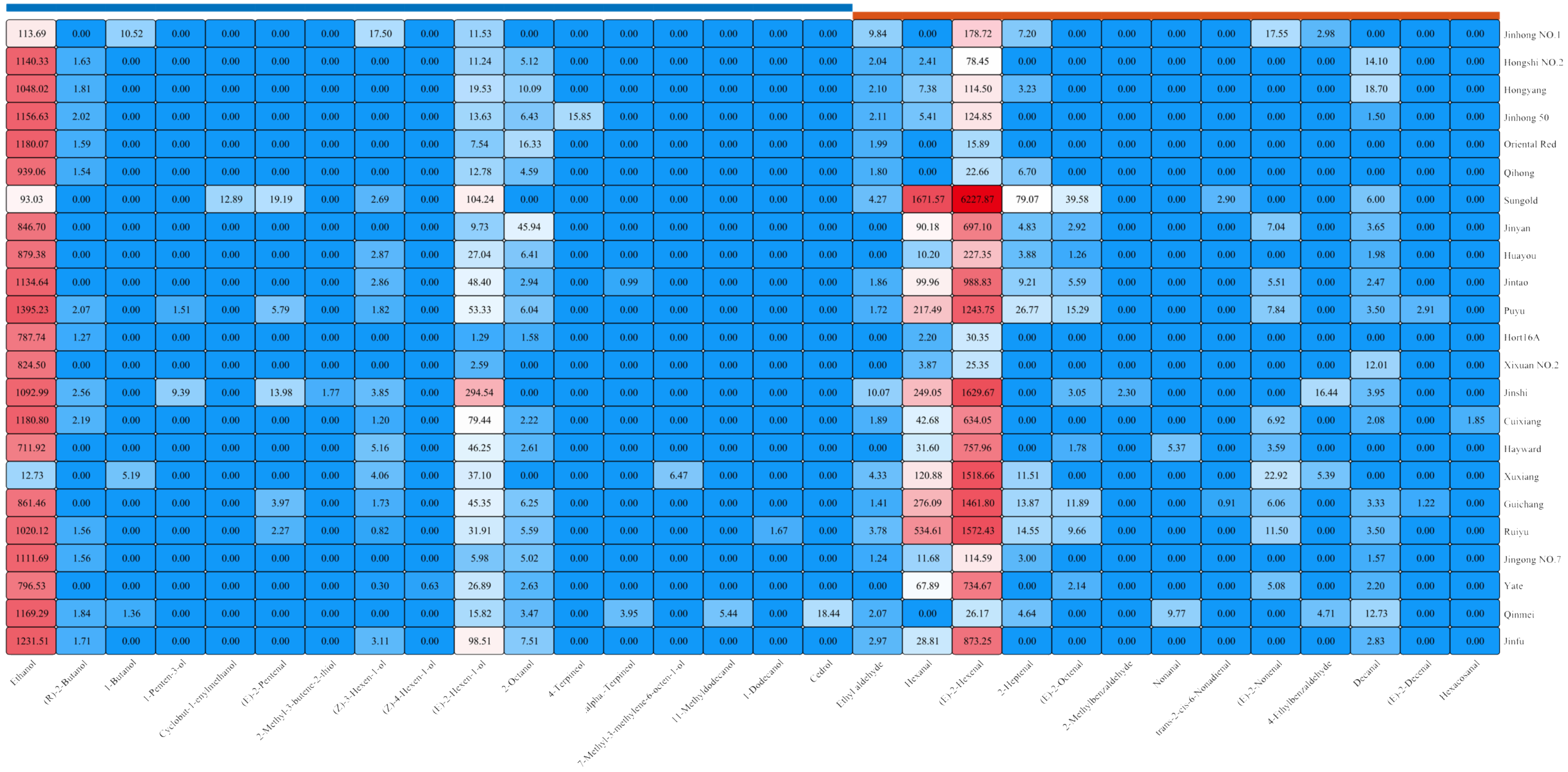

Jinhong NO.1
Hongshi NO.2
Hongyang
Jinhong 50
Oriental Red
Qihong
Sungold
Jinyan
Huayou
Jintao
Puyu
Hort16A
Xixuan NO.2
Jinshi
Cuixiang
Hayward
Xuxiang
Guichang
Ruiyu
Jingong NO.7
Yate
Qinmei
Jinfu
Ethanol
(R)-2-Butanol
1-Butanol
1-Penten-3-ol
Cyclobut-1-enylmethanol
(E)-2-Pentenal
2-Methyl-3-butene-2-thiol
(Z)-3-Hexen-1-ol
(Z)-4-Hexen-1-ol
(E)-2-Hexen-1-ol
2-Octanol
4-Terpineol
.alpha.-Terpineol
7-Methyl-3-methylene-6-octen-1-ol
11-Methyldodecanol
1-Dodecanol
Cedrol
Ethyl aldehyde
Hexanal
(E)-2-Hexenal
2-Heptenal
(E)-2-Octenal
2-Methylbenzaldehyde
Nonanal
trans-2-cis-6-Nonadienal
(E)-2-Nonenal
4-Ethylbenzaldehyde
Decanal
(E)-2-Decenal
Hexacosanal

(B)

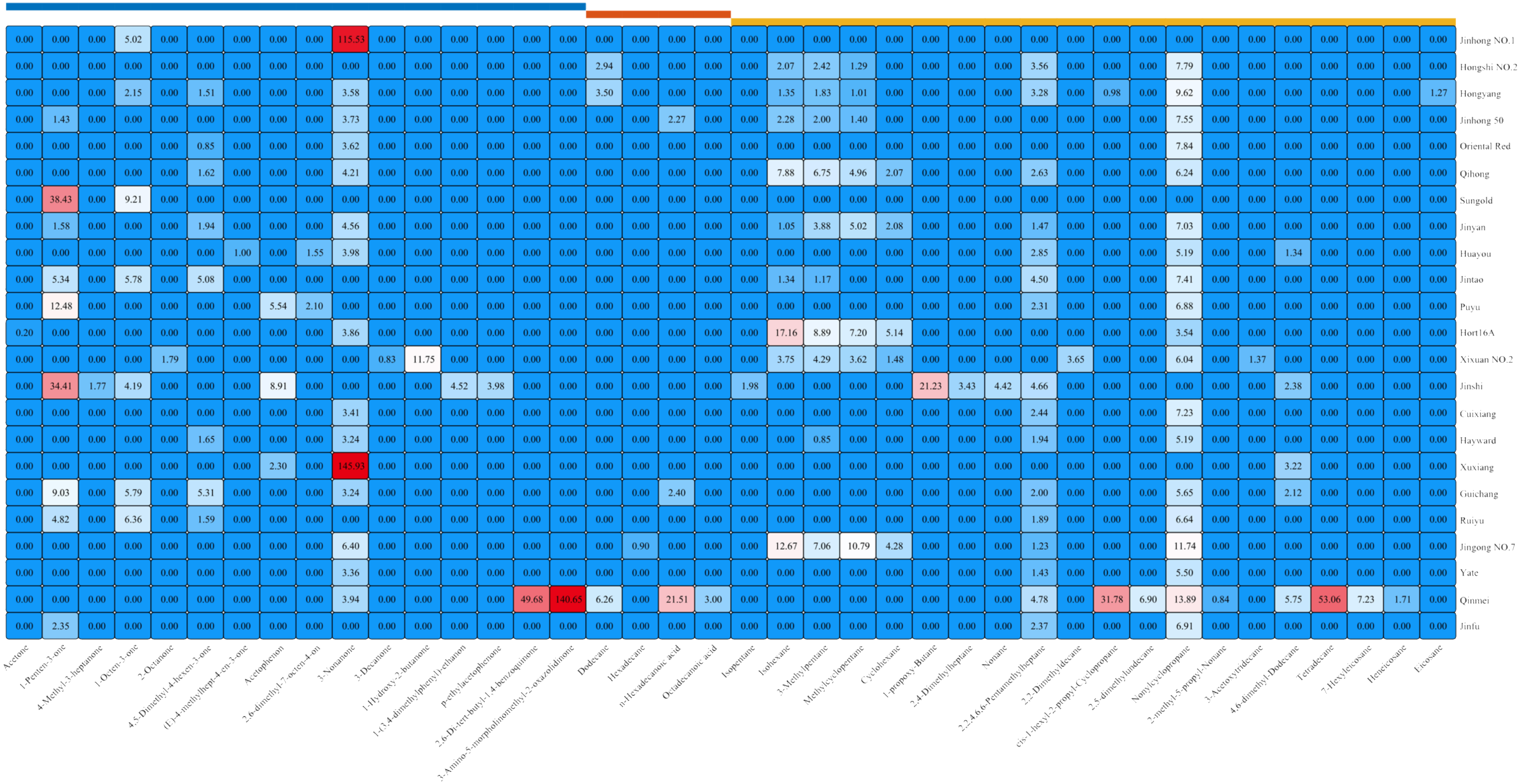
Acetone
1-Penten-3-one
4-Methyl-3-heptanone
1-Octen-3-one
2-Octanone
4,5-Dimethyl-4-hexen-3-one
(E)-4-methylhept-4-en-3-one
Acetophenon
2,6-dimethyl-7-octen-4-on
3-Nonanone
3-Decanone
1-Hydroxy-2-butanone
1-(3,4-dimethylphenyl)-ethanon
p-ethylacetophenone
2,6-Di-tert-butyl-1,4-benzoquinone
3-Amino-5-morpholinomethyl-2-oxazolidinone
Dodecane
Hexadecane
n-Hexadecanoic acid
Octadecanoic acid
Isopentane
Isohexane
3-Methylpentane
Methylcyclopentane
Cyclohexane
1-propoxy-Butane
2,4-Dimethylheptane
Nonane
2,2,4,6,6-Pentamethylheptane
2,2-Dimethyldecane
cis-1-hexyl-2-propyl-Cyclopropane
2,5-dimethylundecane
Nonylcyclopropane
2-methyl-5-propyl-Nonane
3-Acetoxytridecane
4,6-dimethyl-Dodecane
Tetradecane
7-Hexyleicosane
Heneicosane
Eicosane
Jinhong NO.1
Hongshi NO.2
Hongyang
Jinhong 50
Oriental Red
Qihong
Sungold
Jinyan
Huayou
Jintao
Puyu
Hort16A
Xixuan NO.2
Jinshi
Cuixiang
Hayward
Xuxiang
Guichang
Ruiyu
Jingong NO.7
Yate
Qinmei
Jinfu

(C)

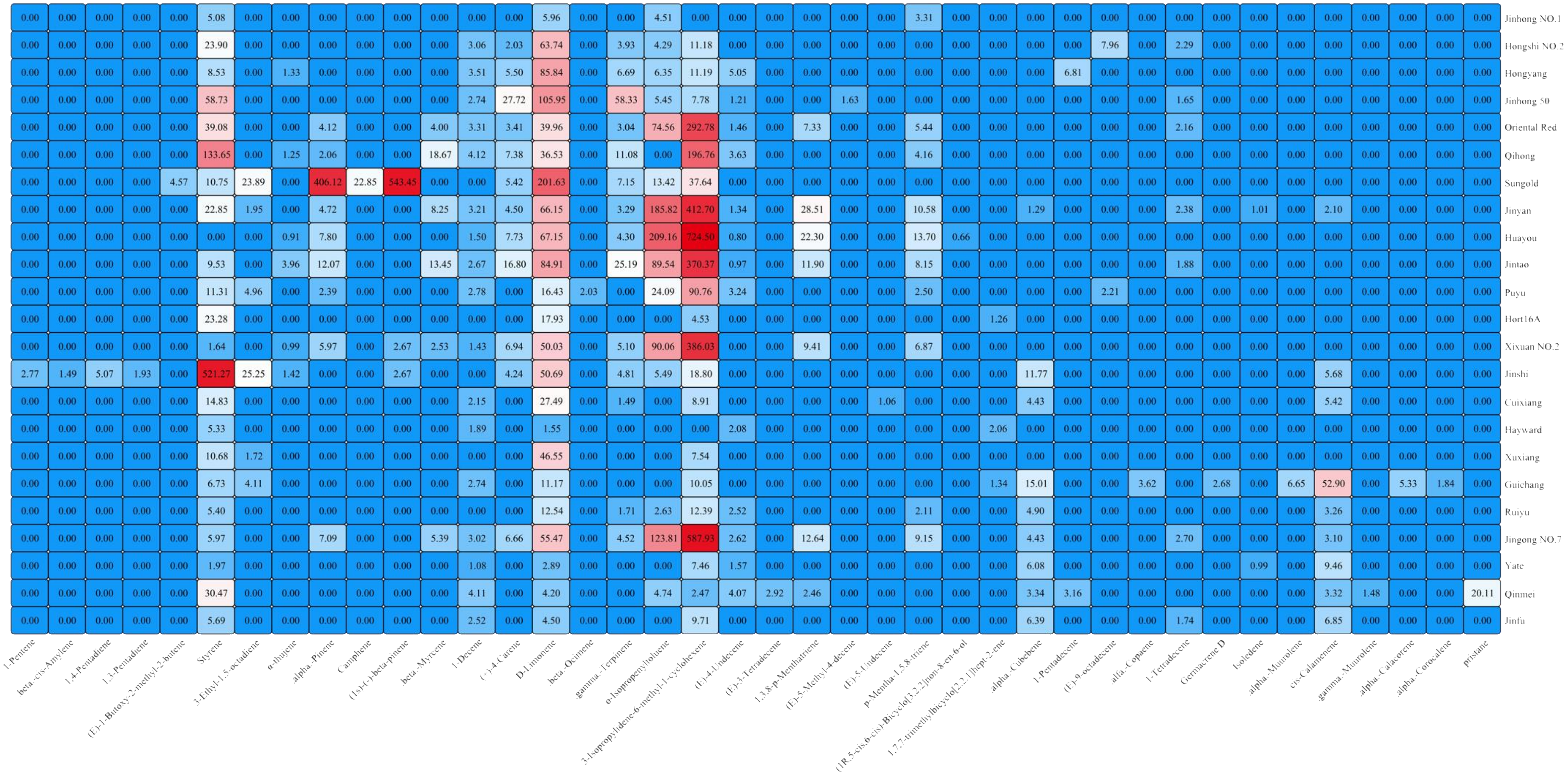

0.00 0.00 0.00 0.00 0.00 5.08 0.00 0.00 0.00 0.00 0.00 0.00 0.00 0.00 5.96 0.00 0.00 4.51 0.00 0.00 0.00 0.00 0.00 0.00 3.31 0.00 0.00 0.00 0.00 0.00 0.00 0.00 0.00 0.00 0.00 0.00 0.00 0.00 0.00 0.00 Jinhong NO.1
0.00 0.00 0.00 0.00 0.00 23.90 0.00 0.00 0.00 0.00 0.00 0.00 3.06 2.03 63.74 0.00 3.93 4.29 11.18 0.00 0.00 0.00 0.00 0.00 0.00 0.00 0.00 0.00 0.00 7.96 0.00 2.29 0.00 0.00 0.00 0.00 0.00 0.00 0.00 0.00 Hongshi NO.2
0.00 0.00 0.00 0.00 0.00 8.53 0.00 1.33 0.00 0.00 0.00 0.00 3.51 5.50 85.84 0.00 6.69 6.35 11.19 5.05 0.00 0.00 0.00 0.00 0.00 0.00 0.00 0.00 6.81 0.00 0.00 0.00 0.00 0.00 0.00 0.00 0.00 0.00 0.00 0.00 Hongyang
0.00 0.00 0.00 0.00 0.00 58.73 0.00 0.00 0.00 0.00 0.00 0.00 2.74 27.72 105.95 0.00 58.33 5.45 7.78 1.21 0.00 0.00 1.63 0.00 0.00 0.00 0.00 0.00 0.00 0.00 0.00 1.65 0.00 0.00 0.00 0.00 0.00 0.00 0.00 0.00 Jinhong 50
0.00 0.00 0.00 0.00 0.00 39.08 0.00 0.00 4.12 0.00 0.00 4.00 3.31 3.41 39.96 0.00 3.04 74.56 292.78 1.46 0.00 7.33 0.00 0.00 5.44 0.00 0.00 0.00 0.00 0.00 0.00 2.16 0.00 0.00 0.00 0.00 0.00 0.00 0.00 0.00 Oriental Red
0.00 0.00 0.00 0.00 0.00 133.65 0.00 1.25 2.06 0.00 0.00 18.67 4.12 7.38 36.53 0.00 11.08 0.00 196.76 3.63 0.00 0.00 0.00 0.00 4.16 0.00 0.00 0.00 0.00 0.00 0.00 0.00 0.00 0.00 0.00 0.00 0.00 0.00 0.00 0.00 Qihong
0.00 0.00 0.00 0.00 4.57 10.75 23.89 0.00 406.12 22.85 543.45 0.00 0.00 5.42 201.63 0.00 7.15 13.42 37.64 0.00 0.00 0.00 0.00 0.00 0.00 0.00 0.00 0.00 0.00 0.00 0.00 0.00 0.00 0.00 0.00 0.00 0.00 0.00 0.00 0.00 Sungold
0.00 0.00 0.00 0.00 0.00 22.85 1.95 0.00 4.72 0.00 0.00 8.25 3.21 4.50 66.15 0.00 3.29 185.82 412.70 1.34 0.00 28.51 0.00 0.00 10.58 0.00 0.00 1.29 0.00 0.00 0.00 2.38 0.00 1.01 0.00 2.10 0.00 0.00 0.00 0.00 Jinyan
0.00 0.00 0.00 0.00 0.00 0.00 0.00 0.91 7.80 0.00 0.00 0.00 1.50 7.73 67.15 0.00 4.30 209.16 724.50 0.80 0.00 22.30 0.00 0.00 13.70 0.66 0.00 0.00 0.00 0.00 0.00 0.00 0.00 0.00 0.00 0.00 0.00 0.00 0.00 0.00 Huayou
0.00 0.00 0.00 0.00 0.00 9.53 0.00 3.96 12.07 0.00 0.00 13.45 2.67 16.80 84.91 0.00 25.19 89.54 370.37 0.97 0.00 11.90 0.00 0.00 8.15 0.00 0.00 0.00 0.00 0.00 0.00 1.88 0.00 0.00 0.00 0.00 0.00 0.00 0.00 0.00 Jintao
0.00 0.00 0.00 0.00 0.00 11.31 4.96 0.00 2.39 0.00 0.00 0.00 2.78 0.00 16.43 2.03 0.00 24.09 90.76 3.24 0.00 0.00 0.00 0.00 2.50 0.00 0.00 0.00 0.00 2.21 0.00 0.00 0.00 0.00 0.00 0.00 0.00 0.00 0.00 0.00 Puyu
0.00 0.00 0.00 0.00 0.00 23.28 0.00 0.00 0.00 0.00 0.00 0.00 0.00 0.00 17.93 0.00 0.00 0.00 4.53 0.00 0.00 0.00 0.00 0.00 0.00 0.00 1.26 0.00 0.00 0.00 0.00 0.00 0.00 0.00 0.00 0.00 0.00 0.00 0.00 0.00 Hort16A
0.00 0.00 0.00 0.00 0.00 1.64 0.00 0.99 5.97 0.00 2.67 2.53 1.43 6.94 50.03 0.00 5.10 90.06 386.03 0.00 0.00 9.41 0.00 0.00 6.87 0.00 0.00 0.00 0.00 0.00 0.00 0.00 0.00 0.00 0.00 0.00 0.00 0.00 0.00 0.00 Xixuan NO.2
2.77 1.49 5.07 1.93 0.00 521.27 25.25 1.42 0.00 0.00 2.67 0.00 0.00 4.24 50.69 0.00 4.81 5.49 18.80 0.00 0.00 0.00 0.00 0.00 0.00 0.00 0.00 11.77 0.00 0.00 0.00 0.00 0.00 0.00 0.00 5.68 0.00 0.00 0.00 0.00 Jinshi
0.00 0.00 0.00 0.00 0.00 14.83 0.00 0.00 0.00 0.00 0.00 0.00 2.15 0.00 27.49 0.00 1.49 0.00 8.91 0.00 0.00 0.00 0.00 1.06 0.00 0.00 0.00 4.43 0.00 0.00 0.00 0.00 0.00 0.00 0.00 5.42 0.00 0.00 0.00 0.00 Cuixiang
0.00 0.00 0.00 0.00 0.00 5.33 0.00 0.00 0.00 0.00 0.00 0.00 1.89 0.00 1.55 0.00 0.00 0.00 0.00 2.08 0.00 0.00 0.00 0.00 0.00 0.00 2.06 0.00 0.00 0.00 0.00 0.00 0.00 0.00 0.00 0.00 0.00 0.00 0.00 0.00 Hayward
0.00 0.00 0.00 0.00 0.00 10.68 1.72 0.00 0.00 0.00 0.00 0.00 0.00 0.00 46.55 0.00 0.00 0.00 7.54 0.00 0.00 0.00 0.00 0.00 0.00 0.00 0.00 0.00 0.00 0.00 0.00 0.00 0.00 0.00 0.00 0.00 0.00 0.00 0.00 0.00 Xuxiang
0.00 0.00 0.00 0.00 0.00 6.73 4.11 0.00 0.00 0.00 0.00 0.00 2.74 0.00 11.17 0.00 0.00 0.00 10.05 0.00 0.00 0.00 0.00 0.00 0.00 0.00 1.34 15.01 0.00 0.00 3.62 0.00 2.68 0.00 6.65 52.90 0.00 5.33 1.84 0.00 Guichang
0.00 0.00 0.00 0.00 0.00 5.40 0.00 0.00 0.00 0.00 0.00 0.00 0.00 0.00 12.54 0.00 1.71 2.63 12.39 2.52 0.00 0.00 0.00 0.00 2.11 0.00 0.00 4.90 0.00 0.00 0.00 0.00 0.00 0.00 0.00 3.26 0.00 0.00 0.00 0.00 Ruiyu
0.00 0.00 0.00 0.00 0.00 5.97 0.00 0.00 7.09 0.00 0.00 5.39 3.02 6.66 55.47 0.00 4.52 123.81 587.93 2.62 0.00 12.64 0.00 0.00 9.15 0.00 0.00 4.43 0.00 0.00 0.00 2.70 0.00 0.00 0.00 3.10 0.00 0.00 0.00 0.00 Jingong NO.7
0.00 0.00 0.00 0.00 0.00 1.97 0.00 0.00 0.00 0.00 0.00 0.00 1.08 0.00 2.89 0.00 0.00 0.00 7.46 1.57 0.00 0.00 0.00 0.00 0.00 0.00 0.00 6.08 0.00 0.00 0.00 0.00 0.00 0.99 0.00 9.46 0.00 0.00 0.00 0.00 Yate
0.00 0.00 0.00 0.00 0.00 30.47 0.00 0.00 0.00 0.00 0.00 0.00 4.11 0.00 4.20 0.00 0.00 4.74 2.47 4.07 2.92 2.46 0.00 0.00 0.00 0.00 0.00 3.34 3.16 0.00 0.00 0.00 0.00 0.00 0.00 3.32 1.48 0.00 0.00 20.11 Qinmei
0.00 0.00 0.00 0.00 0.00 5.69 0.00 0.00 0.00 0.00 0.00 0.00 2.52 0.00 4.50 0.00 0.00 0.00 9.71 0.00 0.00 0.00 0.00 0.00 0.00 0.00 0.00 6.39 0.00 0.00 0.00 1.74 0.00 0.00 0.00 6.85 0.00 0.00 0.00 0.00 Jinfu
1-Pentene
beta.-cis-Amylene
1,4-Pentadiene
1,3-Pentadiene
(E)-1-Butoxy-2-methyl-2-butene
Styrene
3-Ethyl-1,5-octadiene
α-thujene
.alpha.-Pinene
Camphene
(1s)-(-)-beta-pinene
beta.-Myrcene
1-Decene
(+)-4-Carene
D-Limonene
beta.-Ocimene
gamma.-Terpinene
o-Isopropenyltoluene
3-Isopropylidene-6-methyl-1-cyclohexene
(E)-4-Undecene
(E)-3-Tetradecene
1,3,8-p-Menthatriene
(E)-5-Methyl-4-decene
(E)-5-Undecene
p-Mentha-1,5,8-triene
(1R,5-cis,6-cis)-Bicyclo[3.2.2]non-8-en-6-ol
1,7,7-trimethylbicyclo[2.2.1]hept-2-ene
.alpha.-Cubebene
1-Pentadecene
(E)-9-octadecene
alfa.-Copaene
1-Tetradecene
Germacrene D
Isoledene
alpha.-Muurolene
cis-Calamenene
gamma.-Muurolene
.alpha.-Calacorene
.alpha.-Corocalene
pristane

(D)

Sample	Toluene	o-Xylene	p-Xylene	2-Phenylpropene	p-Cymene	n-Butylbenzene	p-propyltoluene	6-methyltetralin	(-)-g-Cadinene	beta-Cadinene	3,5-bis(1,1-dimethylethyl)-pheno	Calamenene	1-Isopropyl-4,7-dimethyl-1,2,3,5,6,8a-hexahydronaphthalene
Jinhong NO.1	5.99	0.00	0.00	0.00	3.42	0.00	0.00	0.00	0.00	0.00	9.94	0.00	0.00
Hongshi NO.2	2.38	7.41	0.00	0.00	18.17	1.56	0.00	0.00	0.00	0.00	2.34	0.00	0.00
Hongyang	2.48	1.88	0.00	0.00	24.78	1.26	2.11	0.00	0.00	0.00	2.81	0.00	0.00
Jinhong 50	14.47	6.95	0.00	0.00	26.43	0.00	0.00	0.00	0.00	0.00	2.48	0.00	0.00
Oriental Red	12.35	7.87	0.00	0.00	17.53	1.75	1.74	0.00	0.00	0.00	2.16	0.00	0.00
Qihong	1.31	20.12	39.14	0.00	29.61	0.00	1.49	0.00	0.00	0.00	2.20	0.00	0.00
Sungold	33.40	48.24	0.00	0.00	28.08	0.00	0.00	0.00	0.00	0.00	0.00	0.00	0.00
Jinyan	1.21	18.92	0.00	0.00	22.67	1.03	0.75	0.00	0.00	0.00	2.16	0.00	0.00
Huayou	0.00	0.00	0.00	0.00	32.30	1.52	0.00	0.00	0.00	0.00	1.93	0.00	0.00
Jintao	4.10	10.48	0.00	0.00	47.77	1.30	1.56	0.00	0.00	0.00	1.97	0.00	0.00
Puyu	1.98	11.23	0.00	0.00	5.01	0.00	0.00	0.00	0.00	0.00	2.73	0.00	0.00
Hort16A	6.61	2.55	0.00	0.00	0.00	0.00	0.00	0.00	0.00	0.00	1.80	0.00	0.00
Xixuan NO.2	0.00	0.00	0.00	0.00	14.26	0.00	0.00	0.00	0.00	0.00	1.37	0.00	0.00
Jinshi	38.54	196.84	0.00	2.64	18.55	0.00	0.00	0.00	0.00	0.00	10.85	0.00	0.00
Cuixiang	9.44	7.75	0.00	0.00	3.86	0.00	1.44	0.00	0.00	0.00	2.69	0.00	0.00
Hayward	1.84	5.50	0.00	0.00	0.00	0.00	0.00	0.00	0.00	0.00	1.45	0.00	0.00
Xuxiang	6.58	16.06	0.00	0.00	6.67	0.00	0.00	0.00	0.00	0.00	9.45	0.00	0.00
Guichang	6.72	20.64	0.00	0.00	0.00	0.00	0.00	0.00	6.51	0.00	2.52	0.00	0.00
Ruiyu	1.86	22.71	0.00	0.00	5.99	0.00	1.38	0.00	0.00	0.00	2.20	0.00	0.00
Jingong NO.7	1.26	2.86	4.04	0.00	25.59	2.12	1.70	0.00	0.00	1.95	2.11	10.05	4.17
Yate	0.00	6.87	0.00	0.00	0.00	0.00	0.00	0.00	0.00	0.00	2.07	0.00	0.00
Qinmei	0.82	10.11	0.00	0.00	1.73	2.16	1.05	1.42	0.00	0.00	19.68	0.00	0.00
Jinfu	0.00	4.80	0.00	0.00	1.51	0.00	0.00	0.00	0.00	0.00	2.48	0.00	0.00

Sample	1,6-Dimethyl-4-(1-methylethyl)-(1,2,3,4,4a,7)hexahydronaphthalene	2,6-di-tert-Butyl-4-sec-butylphenol	Cadalene	Ammonium carbamate	Aether	Methylsulfide	Ammonium acetate	Propionic anhydride	Methylterahydropyran	Oxime-, methoxy-phenyl-_	Anisole	4-Octadecylmorpholine
Jinhong NO.1	0.00	0.00	0.00	11.45	0.00	0.00	0.00	24.21	0.00	121.22	0.00	0.00
Hongshi NO.2	0.00	0.00	0.00	7.70	0.39	2.70	0.00	0.00	0.00	117.74	0.00	0.00
Hongyang	0.00	0.00	0.00	7.82	0.28	1.53	0.00	4.84	0.00	127.49	0.00	0.00
Jinhong 50	0.00	0.00	0.00	9.95	1.96	3.91	0.00	5.91	0.00	125.01	0.00	0.00
Oriental Red	0.00	0.00	0.00	7.56	1.14	0.00	0.00	7.69	0.00	99.85	0.00	0.00
Qihong	0.00	0.00	0.00	6.95	0.94	1.90	0.00	5.25	0.00	100.31	0.00	0.00
Sungold	0.00	0.00	0.00	18.21	0.00	4.63	0.00	0.00	0.00	291.96	0.00	0.00
Jinyan	0.00	0.00	0.00	7.21	0.50	2.75	0.00	5.58	0.00	106.68	1.80	0.00
Huayou	0.00	0.00	0.00	7.66	0.82	3.56	0.00	5.06	0.00	71.72	0.00	0.00
Jintao	0.00	0.00	0.00	8.67	0.82	0.00	0.00	0.00	0.00	109.51	0.00	0.00
Puyu	0.00	0.00	0.00	11.78	1.66	0.00	0.00	7.47	0.00	155.53	0.00	0.00
Hort16A	0.00	0.00	0.00	4.42	0.65	1.51	0.00	4.31	0.00	60.51	0.00	0.00
Xixuan NO.2	0.00	0.00	0.00	5.52	0.79	1.71	0.00	3.80	0.00	53.75	0.00	0.00
Jinshi	0.00	0.00	0.00	28.61	9.33	0.00	0.00	16.49	2.23	116.66	0.00	0.00
Cuixiang	0.00	0.00	0.00	9.21	1.24	0.00	0.00	7.15	0.00	103.69	0.00	0.00
Hayward	0.00	0.00	0.00	4.88	0.00	0.00	0.00	3.65	0.00	62.64	0.00	0.00
Xuxiang	0.00	0.00	0.00	13.33	0.00	4.97	3.77	24.14	0.00	152.41	0.00	0.00
Guichang	12.63	0.00	6.07	6.53	0.72	0.00	0.00	0.00	0.00	87.49	0.00	0.00
Ruiyu	0.00	0.00	0.00	9.47	0.34	1.16	0.00	0.00	0.00	189.39	0.00	0.00
Jingong NO.7	2.72	0.00	0.00	6.36	0.88	0.00	0.00	4.38	0.00	79.38	0.00	0.00
Yate	2.52	0.00	1.88	5.08	0.71	0.00	0.00	4.04	0.00	82.94	0.00	0.00
Qinmei	0.00	59.02	0.00	14.10	1.12	2.47	0.00	0.00	0.00	133.34	0.00	20.09
Jinfu	0.00	0.00	0.00	10.60	0.69	0.00	0.00	7.58	0.00	143.59	0.00	0.00

(E)

Figure S1. Quantitative analysis of esters (**A**), alcohols and aldehydes (**B**), ketones, acids and alkanes (**C**), alkenes (**D**) and aromatic hydrocarbons and their homologs and other volatile components (**E**) in kiwifruit samples. The concentration of volatile components gradually increased from blue to red in units of μg/L.

Figure S2

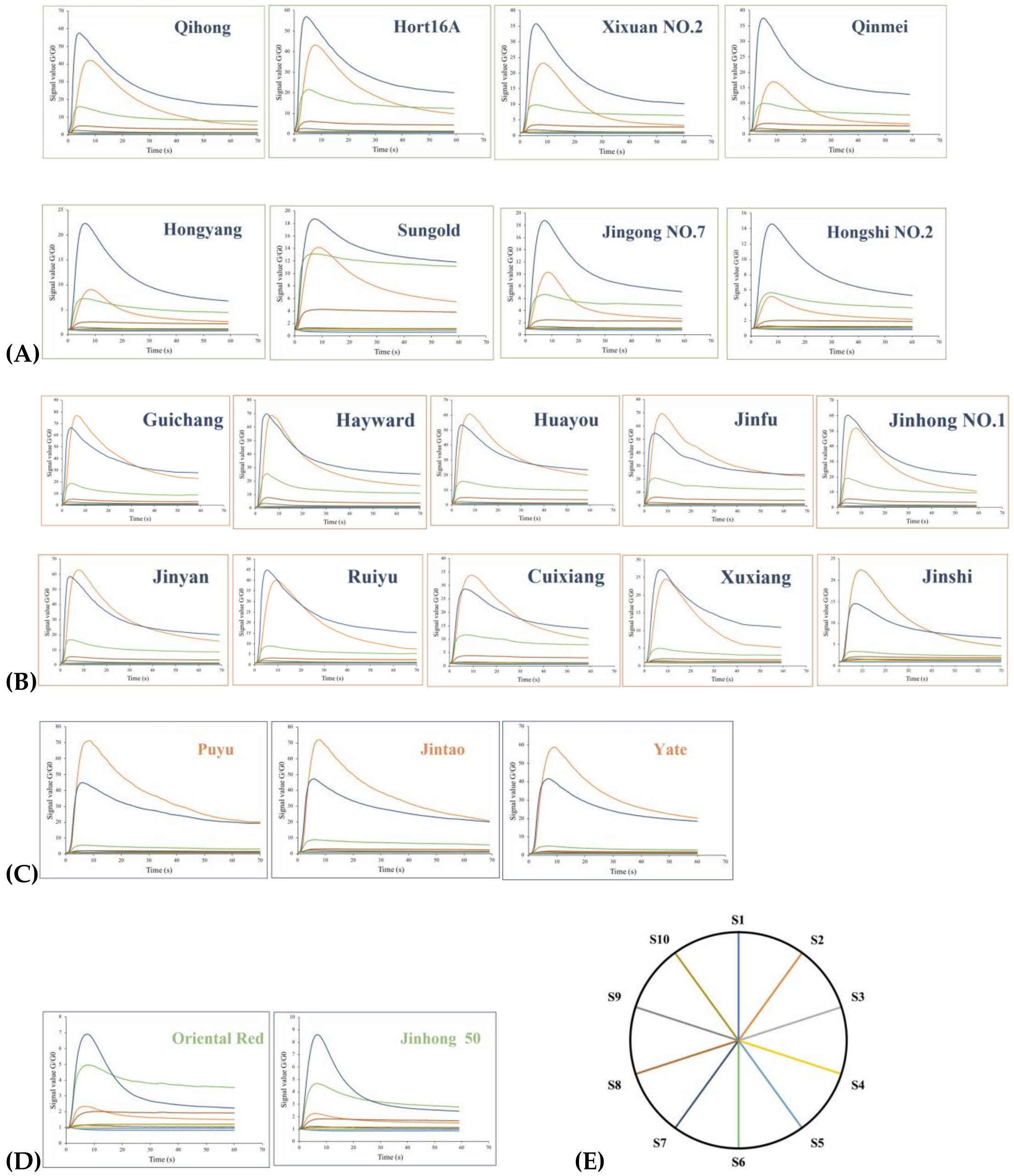

Figure S2. Kiwifruits samples were divided into 4 categories based on electronic nose response (**A**)-(**D**). Electronic nose sensor (**E**).

Table S1. Cultivars, species, flesh color, region and retail price of kiwifruits.

Cultivar	Species	Flesh color	Region	Price (RMB, ¥/Kg) [a]
Jinhong NO.1	*A. chinensis*	Yellow, and red (middle part)	Jiangsu, China	*47*
Hongshi NO.2	*A. chinensis*	Yellow, and red (middle part)	Sichuan, China	*35*
Hongyang	*A. chinensis*	Green, and red (middle part)	Shaanxi, China	26
Jinhong 50	*A. chinensis*	Green, and red (middle part)	Sichuan, China	*47*
Oriental Red	*A. chinensis*	Yellow, and red (middle part)	Shaanxi, China	*26*
Qihong	*A. chinensis*	Green, and red (middle part)	Shaanxi, China	*30*
Sungold	*A. chinensis*	Yellow	New Zealand	*42*
Jinyan	*A. chinensis*	Yellow	Henan, China	*16*
Huayou	*A. chinensis*	Yellow	Shaanxi, China	*14*
Jintao	*A. chinensis*	Yellow	Henan, China	*31*
Puyu	*A. chinensis*	Yellow	Shaanxi, China	*76*
Hort16A	*A. chinensis*	Yellow	Shaanxi, China	*15*
Xixuan NO.2	*A. chinensis*	Yellow	Shaanxi, China	*20*
Jinshi	*A. chinensis*	Yellow	Sichuan, China	*35*
Cuixiang	*A. deliciosa*	Green	Shaanxi, China	*9*
Hayward	*A. deliciosa*	Green	Shaanxi, China	*8*
Xuxiang	*A. deliciosa*	Green	Shaanxi, China	*8*
Guichang	*A. deliciosa*	Green	Shaanxi, China	*42*
Ruiyu	*A. deliciosa*	Green	Shaanxi, China	*78*
Jingong NO.7	*A. chinensis*	Green	Jiangsu, China	*47*
Yate	*A. deliciosa*	Green	Shaanxi, China	*8*
Qinmei	*A. deliciosa*	Green	Shaanxi, China	*8*
Jinfu	*A. deliciosa*	Green	Shaanxi, China	*26*

[a] The retail price was the average price of at least 5 different big chain hypermarkets.

Table S2. The determination of internal standards.

No	Name	CAS	Detected in the samples	Internal quantitative standard
1	(S)-(+)-2-Octanol	6169-06-8	\	√
2	3-Octanol	589-98-0	\	√
3	Adonitol	488-81-3	\	\
4	Cyclohexanone	108-94-1	\	\
5	2-Octanone	111-13-7	√(1)	√
6	3-Octanone	106-68-3	\	√
7	2-Nonanone	821-55-6	\	√
8	3-Nonanone	925-78-0	√ (13)	√
9	Octyl acetate	112-14-1	\	\
10	Ethyl caprylate	106-32-1	\	\
11	Triacetin	102-76-1	\	\

Table S3. The concentration of volatile components in different cultivars of kiwifruit.

Component	Concentration (μg/L) [a]																						
	Jinhong NO.1	Hongshi NO.2	Hongyang	Jinhong 50	Oriental Red	Qihong	Sungold	Jinyan	Huayou	Jintao	Puyu	Hort16A	Xixuan NO.2	Jinshi	Cuixiang	Hayward	Xuxiang	Guichang	Ruiyu	Jingong NO.7	Yate	Qinmei	Jinfu
Esters																							
Methyl acetate	5.89	-	-	-	-	-	-	-	-	-	-	-	-	4.72	-	-	25.56	-	-	-	-	-	-
Ethyl acetate	430.85	8.11	2.08	6.46	3.25	8.73	5.23	18.70	3.74	4.92	4.46	20.53	12.56	40.77	3.29	3.20	286.27	1.42	4.29	29.94	1.66	50.20	10.26
tert-Butyl isopropyl carbonate	-	-	-	-	-	-	-	-	-	-	-	-	-	-	-	-	-	-	-	-	-	3.42	-
Methyl propionate	-	-	-	-	-	-	-	-	-	-	-	-	-	-	-	-	4.14	-	-	-	-	2.22	-
n-Propyl acetate	-	20.71	21.05	21.09	21.95	17.95	-	12.83	14.51	17.01	24.26	14.46	13.33	43.27	22.27	12.24	-	12.99	19.19	17.66	13.17	21.25	21.04
Methyl butanoate	104.07	-	-	3.66	-	-	-	72.30	48.06	-	-	-	18.79	144.68	5.39	-	2675.60	6.19	2.86	4.98	7.60	682.28	197.03
trans-but-2-enoic acid methyl ester	-	-	-	-	-	-	-	-	-	-	-	-	-	-	-	-	2.53	-	-	-	-	-	-
Ethyl isobutyrate	-	-	-	-	-	-	-	-	-	-	-	-	-	-	-	-	2.81	-	-	-	-	20.84	-
sec-Butyl acetate	-	9.58	8.49	10.14	10.44	-	-	6.33	7.24	8.10	11.25	7.29	6.21	15.72	10.38	6.54	-	6.56	8.14	8.35	6.72	-	10.73
Butyl acetate	21.42	-	-	-	-	-	-	-	-	-	-	-	-	-	-	-	29.77	-	-	-	-	2.25	-
Diethyl carbonate	-	-	-	-	3.54	-	-	-	-	-	-	-	-	-	-	-	-	-	-	-	-	-	-
Ethyl butanoate	7042.01	10.86	2.89	132.46	85.59	1.59	149.65	1248.97	967.27	5.43	9.94	18.40	36.19	214.13	34.76	2.14	1561.04	1.56	28.30	4.62	13.25	362.43	80.60
Methyl valerate	-	-	-	-	-	-	-	-	-	-	-	-	-	2.04	-	-	23.31	-	-	-	-	3.72	3.31
Ethyl 2-methylbutanoate	9.62	-	-	-	-	-	-	-	-	-	-	-	-	-	-	-	-	-	-	-	-	2.28	-
Hexyl formate	11.29	3.21	15.27	7.95	-	1.59	142.20	11.07	31.08	51.24	61.79	-	3.23	167.99	74.99	23.08	68.52	56.28	91.89	5.68	23.74	5.49	65.03
n-Propyl butyrate	85.50	-	-	-	-	-	-	-	3.43	-	9.80	-	-	-	-	-	-	-	-	-	-	5.03	-
Heptyl butanoate	-	-	-	-	-	-	-	-	1.36	-	-	-	-	-	-	-	-	-	-	-	-	-	-
Ethyl pentanoate	66.12	-	-	1.63	10.96	-	-	59.85	8.25	-	-	-	-	-	-	-	18.29	-	-	-	5.08	1.71	3.89
Isobutyl isobutyrate	-	-	-	-	-	-	-	-	-	-	-	-	-	-	-	-	-	-	-	-	-	3.86	-

Methyl hexanoate	11.02	-	-	-	-	-	-	5.96	10.47	-	-	-	25.31	7.51	3.55	-	226.78	2.69	2.37	1.46	2.65	94.08	54.85
Ethyl 3-hydroxybutyrate	16.37	-	-	-	-	-	-	-	-	-	-	-	-	-	-	-	-	-	-	-	-	-	-
Butyl butanoate	188.52	-	-	2.23	-	-	-	409.61	101.02	-	-	-	82.78	-	-	-	103.24	-	-	-	-	17.28	3.57
Methyl 2-hexenoate	-	-	-	-	-	-	-	-	-	-	-	-	-	-	-	-	2.10	-	-	-	-	-	-
Ethyl hexanoate	1223.14	11.98	9.05	28.98	39.50	10.94	25.56	382.02	438.54	20.60	18.08	9.32	8.82	-	16.01	8.59	52.17	15.68	24.31	10.31	7.54	36.59	33.92
Methyl 3-(methylthio)propionate	-	-	-	-	-	-	-	-	-	-	-	-	-	-	-	-	25.71	-	-	-	-	-	-
trans-. beta. - Ocimene	-	-	-	-	-	-	-	-	-	-	3.81	-	-	2.18	-	-	-	-	-	-	-	-	-
Methyl 2-ethylhexanoate	-	-	-	-	-	-	-	-	1.32	-	-	-	-	-	-	-	-	-	-	-	-	-	-
Methyl benzoate	-	4.82	4.16	2.52	-	53.80	-	64.94	25.98	95.24	2.64	-	-	-	4.31	-	104.95	1.63	4.30	14.86	1.69	69.56	17.97
Ethyl benzoate	349.12	-	-	4.55	-	-	-	188.64	8.02	-	-	-	-	-	2.43	-	43.37	-	-	-	-	9.37	13.29
Butyl hexanoate	5.46	-	-	-	7.53	-	-	15.87	6.47	4.39	12.69	-	22.84	-	-	-	2.50	-	-	7.69	1.73	-	-
Ethyl octanoate	-	-	-	-	1.70	-	5.00	9.87	64.28	-	-	-	-	1.63	1.45	-	-	1.30	2.31	2.28	-	3.96	3.22
Sulfurous acid, 2-ethylhexyl nonyl ester	-	-	-	-	-	-	-	-	-	-	-	-	-	-	-	-	-	-	-	-	-	4.72	-
Butyl benzoate	5.50	-	-	-	-	-	-	32.36	-	-	-	-	8.04	-	-	-	5.42	-	-	-	-	-	-
Ethyl caprate	-	-	-	-	-	-	14.15	-	3.23	-	-	-	-	-	-	-	-	-	-	-	-	-	-
2,2,4-Trimethyl-1,3-pentanediol diisobutyrate	-	-	-	-	-	-	-	-	-	-	-	-	-	-	-	-	-	-	-	-	-	36.52	-
Diisobutyl phthalate	-	4.29	4.36	4.35	6.43	4.23	-	3.73	4.91	5.12	7.13	3.40	6.56	3.19	6.20	5.15	-	4.27	5.73	8.14	2.70	8.78	5.61
Dibutyl phthalate	-	5.86	4.03	4.24	6.53	6.35	-	3.19	7.55	4.06	5.13	4.19	6.16	-	8.51	3.95	-	6.79	6.23	4.74	3.85	15.77	6.75
Alcohols																							
Ethanol	113.69	1140.33	1048.02	1156.63	1180.07	939.06	93.03	846.70	879.38	1134.64	1395.23	787.74	824.50	1092.99	1180.80	711.92	12.73	861.46	1020.12	1111.69	796.53	1169.29	1231.51
2-Butanol, (R)-	-	1.63	1.81	2.02	1.59	1.54	-	-	-	-	2.07	1.27	-	2.56	2.19	-	-	-	1.56	1.56	-	1.84	1.71
1-Butanol	10.52	-	-	-	-	-	-	-	-	-	-	-	-	-	-	-	5.19	-	-	-	-	1.36	-
1-Penten-3-ol	-	-	-	-	-	-	-	-	-	-	1.51	-	-	9.39	-	-	-	-	-	-	-	-	-

Cyclobut-1-enylmethanol	-	-	-	-	-	-	12.89	-	-	-	-	-	-	-	-	-	-	-	-	-	-	-	-
2-Pentenal, (E)-	-	-	-	-	-	-	19.19	-	-	-	5.79	-	-	13.98	-	-	-	3.97	2.27	-	-	-	-
2-Methyl-3-butene-2-thiol	-	-	-	-	-	-	-	-	-	-	-	-	-	1.77	-	-	-	-	-	-	-	-	-
3-Hexen-1-ol, (Z)-	17.50	-	-	-	-	-	2.69	-	2.87	2.86	1.82	-	-	3.85	1.20	5.16	4.06	1.73	0.82	-	0.30	-	3.11
4-Hexen-1-ol, (Z)-	-	-	-	-	-	-	-	-	-	-	-	-	-	-	-	-	-	-	-	-	0.63	-	-
2-Hexen-1-ol, (E)-	11.53	11.24	19.53	13.63	7.54	12.78	104.24	9.73	27.04	48.40	53.33	1.29	2.59	294.54	79.44	46.25	37.10	45.35	31.91	5.98	26.89	15.82	98.51
2-Octanol	-	5.12	10.09	6.43	16.33	4.59	-	45.94	6.41	2.94	6.04	1.58	-	-	2.22	2.61	-	6.25	5.59	5.02	2.63	3.47	7.51
Terpinen-4-ol	-	-	-	15.85	-	-	-	-	-	-	-	-	-	-	-	-	-	-	-	-	-	-	-
.alpha.-Terpineol	-	-	-	-	-	-	-	-	-	0.99	-	-	-	-	-	-	-	-	-	-	-	3.95	-
6-Octen-1-ol, 7-methyl-3-methylene-	-	-	-	-	-	-	-	-	-	-	-	-	-	-	-	-	6.47	-	-	-	-	-	-
11-Methyldodecanol	-	-	-	-	-	-	-	-	-	-	-	-	-	-	-	-	-	-	-	-	-	5.44	-
1-Dodecanol	-	-	-	-	-	-	-	-	-	-	-	-	-	-	-	-	-	-	1.67	-	-	-	-
Cedrol	-	-	-	-	-	-	-	-	-	-	-	-	-	-	-	-	-	-	-	-	-	18.44	-
Aldehydes																							
Acetaldehyde	9.84	2.04	2.10	2.11	1.99	1.80	4.27	-	-	1.86	1.72	-	-	10.07	1.89	-	4.33	1.41	3.78	1.24	-	2.07	2.97
Hexanal	-	2.41	7.38	5.41	-	-	1671.57	90.18	10.20	99.96	217.49	2.20	3.87	249.05	42.68	31.60	120.88	276.09	534.61	11.68	67.89	-	28.81
2-Hexenal, (E)-	178.72	78.45	114.50	124.85	15.89	22.66	6227.87	697.10	227.35	988.83	1243.75	30.35	25.35	1629.67	634.05	757.96	1518.66	1461.80	1572.43	114.59	734.67	26.17	873.25
2-Heptenal, (Z)-	7.20	-	3.23	-	-	6.70	79.07	4.83	3.88	9.21	26.77	-	-	-	-	-	11.51	13.87	14.55	3.00	-	4.64	-
2-Octenal, (E)-	-	-	-	-	-	-	39.58	2.92	1.26	5.59	15.29	-	-	3.05	-	1.78	-	11.89	9.66	-	2.14	-	-
Benzaldehyde, 2-methyl-	-	-	-	-	-	-	-	-	-	-	-	-	-	2.30	-	-	-	-	-	-	-	-	-
Nonanal	-	-	-	-	-	-	-	-	-	-	-	-	-	-	-	5.37	-	-	-	-	-	9.77	-
2,6-Nonadienal, (E,Z)-	-	-	-	-	-	-	2.90	-	-	-	-	-	-	-	-	-	-	0.91	-	-	-	-	-
2-Nonenal, (E)-	17.55	-	-	-	-	-	-	7.04	-	5.51	7.84	-	-	-	6.92	3.59	22.92	6.06	11.50	-	5.08	-	-
Benzaldehyde, 4-ethyl-	2.98	-	-	-	-	-	-	-	-	-	-	-	-	16.44	-	-	5.39	-	-	-	-	4.71	-
Decanal	-	14.10	18.70	1.50	-	-	6.00	3.65	1.98	2.47	3.50	-	12.01	3.95	2.08	-	-	3.33	3.50	1.57	2.20	12.73	2.83
2-Decenal, (E)-	-	-	-	-	-	-	-	-	-	-	2.91	-	-	-	-	-	-	1.22	-	-	-	-	-

Hexacosanal	-	-	-	-	-	-	-	-	-	-	-	-	-	-	1.85	-	-	-	-	-	-	-	-
Ketones																							
Acetone	-	-	-	-	-	-	-	-	-	-	-	0.20	-	-	-	-	-	-	-	-	-	-	-
1-Penten-3-one	-	-	-	1.43	-	-	38.43	1.58	-	5.34	12.48	-	-	34.41	-	-	-	9.03	4.82	-	-	-	2.35
3-Heptanone, 4-methyl-	-	-	-	-	-	-	-	-	-	-	-	-	-	1.77	-	-	-	-	-	-	-	-	-
1-Octen-3-one	5.02	-	2.15	-	-	-	9.21	-	-	5.78	-	-	-	4.19	-	-	-	5.79	6.36	-	-	-	-
2-Octanone	-	-	-	-	-	-	-	-	-	-	-	-	1.79	-	-	-	-	-	-	-	-	-	-
4-Hexen-3-one, 4,5-dimethyl-	-	-	1.51	-	0.85	1.62	-	1.94	-	5.08	-	-	-	-	-	1.65	-	5.31	1.59	-	-	-	-
4-Hepten-3-one, 4-methyl-	-	-	-	-	-	-	-	-	1.00	-	-	-	-	-	-	-	-	-	-	-	-	-	-
Acetophenone	-	-	-	-	-	-	-	-	-	-	5.54	-	-	8.91	-	-	2.30	-	-	-	-	-	-
7-Octen-4-one, 2,6-dimethyl-	-	-	-	-	-	-	-	-	1.55	-	2.10	-	-	-	-	-	-	-	-	-	-	-	-
3-Nonanone	115.53	-	3.58	3.73	3.62	4.21	-	4.56	3.98	-	-	3.86	-	-	3.41	3.24	145.93	3.24	-	6.40	3.36	3.94	-
3-Decanone	-	-	-	-	-	-	-	-	-	-	-	-	0.83	-	-	-	-	-	-	-	-	-	-
1-Hydroxy-2-butanone	-	-	-	-	-	-	-	-	-	-	-	-	11.75	-	-	-	-	-	-	-	-	-	-
Ethanone, 1-(3,4-dimethylphenyl)-	-	-	-	-	-	-	-	-	-	-	-	-	-	4.52	-	-	-	-	-	-	-	-	-
Ethanone, 1-(4-ethylphenyl)-	-	-	-	-	-	-	-	-	-	-	-	-	-	3.98	-	-	-	-	-	-	-	-	-
2,5-Cyclohexadiene-1,4-dione, 2,6-bis(1,1-dimethylethyl)-	-	-	-	-	-	-	-	-	-	-	-	-	-	-	-	-	-	-	-	-	-	49.68	-
2-Oxazolidinone, 3-amino-5-(4-morpholinylmethyl)-	-	-	-	-	-	-	-	-	-	-	-	-	-	-	-	-	-	-	-	-	-	140.65	-
Acids																							
Dodecane	-	2.94	3.50	-	-	-	-	-	-	-	-	-	-	-	-	-	-	-	-	-	-	6.26	-
Hexadecane	-	-	-	-	-	-	-	-	-	-	-	-	-	-	-	-	-	-	-	0.90	-	-	-
n-Hexadecanoic acid	-	-	-	2.27	-	-	-	-	-	-	-	-	-	-	-	-	-	2.40	-	-	-	21.51	-
Octadecanoic acid	-	-	-	-	-	-	-	-	-	-	-	-	-	-	-	-	-	-	-	-	-	3.00	-

Alkanes																							
Butane, 2-methyl-	-	-	-	-	-	-	-	-	-	-	-	-	-	1.98	-	-	-	-	-	-	-	-	-
Pentane, 2-methyl-	-	2.07	1.35	2.28	-	7.88	-	1.05	-	1.34	-	17.16	3.75	-	-	-	-	-	-	12.67	-	-	-
Pentane, 3-methyl-	-	2.42	1.83	2.00	-	6.75	-	3.88	-	1.17	-	8.89	4.29	-	-	0.85	-	-	-	7.06	-	-	-
Cyclopentane, methyl-	-	1.29	1.01	1.40	-	4.96	-	5.02	-	-	-	7.20	3.62	-	-	-	-	-	-	10.79	-	-	-
Cyclohexane	-	-	-	-	-	2.07	-	2.08	-	-	-	5.14	1.48	-	-	-	-	-	-	4.28	-	-	-
Butane, 1-propoxy-	-	-	-	-	-	-	-	-	-	-	-	-	-	21.23	-	-	-	-	-	-	-	-	-
Heptane, 2,4-dimethyl-	-	-	-	-	-	-	-	-	-	-	-	-	-	3.43	-	-	-	-	-	-	-	-	-
Nonane	-	-	-	-	-	-	-	-	-	-	-	-	-	4.42	-	-	-	-	-	-	-	-	-
Heptane, 2,2,4,6,6-pentamethyl-	-	3.56	3.28	-	-	2.63	-	1.47	2.85	4.50	2.31	-	-	4.66	2.44	1.94	-	2.00	1.89	1.23	1.43	4.78	2.37
Decane, 2,2-dimethyl-	-	-	-	-	-	-	-	-	-	-	-	-	3.65	-	-	-	-	-	-	-	-	-	-
Cyclopropane, 1-hexyl-2-propyl-, cis-	-	-	0.98	-	-	-	-	-	-	-	-	-	-	-	-	-	-	-	-	-	-	31.78	-
Undecane, 2,5-dimethyl-	-	-	-	-	-	-	-	-	-	-	-	-	-	-	-	-	-	-	-	-	-	6.90	-
Cyclopropane, nonyl-	-	7.79	9.62	7.55	7.84	6.24	-	7.03	5.19	7.41	6.88	3.54	6.04	-	7.23	5.19	-	5.65	6.64	11.74	5.50	13.89	6.91
Nonane, 2-methyl-5-propyl-	-	-	-	-	-	-	-	-	-	-	-	-	-	-	-	-	-	-	-	-	-	0.84	-
3-Acetoxytridecane	-	-	-	-	-	-	-	-	-	-	-	-	1.37	-	-	-	-	-	-	-	-	-	-
Dodecane, 4,6-dimethyl-	-	-	-	-	-	-	-	-	1.34	-	-	-	-	2.38	-	-	3.22	2.12	-	-	-	5.75	-
Tetradecane	-	-	-	-	-	-	-	-	-	-	-	-	-	-	-	-	-	-	-	-	-	53.06	-
Eicosane, 7-hexyl-	-	-	-	-	-	-	-	-	-	-	-	-	-	-	-	-	-	-	-	-	-	7.23	-
Heneicosane	-	-	-	-	-	-	-	-	-	-	-	-	-	-	-	-	-	-	-	-	-	1.71	-
Eicosane	-	-	1.27	-	-	-	-	-	-	-	-	-	-	-	-	-	-	-	-	-	-	-	-
Alkenes																							
1-Pentene	-	-	-	-	-	-	-	-	-	-	-	-	-	2.77	-	-	-	-	-	-	-	-	-
2-Pentene, (Z)-	-	-	-	-	-	-	-	-	-	-	-	-	-	1.49	-	-	-	-	-	-	-	-	-
1,4-Pentadiene	-	-	-	-	-	-	-	-	-	-	-	-	-	5.07	-	-	-	-	-	-	-	-	-
1,3-Pentadiene	-	-	-	-	-	-	-	-	-	-	-	-	-	1.93	-	-	-	-	-	-	-	-	-
1-Butoxy-2-methyl-2-butene (E)-	-	-	-	-	-	-	4.57	-	-	-	-	-	-	-	-	-	-	-	-	-	-	-	-

Styrene	5.08	23.90	8.53	58.73	39.08	133.65	10.75	22.85	-	9.53	11.31	23.28	1.64	521.27	14.83	5.33	10.68	6.73	5.40	5.97	1.97	30.47	5.69
3-Ethyl-1,5-octadiene	-	-	-	-	-	-	23.89	1.95	-	-	4.96	-	-	25.25	-	-	1.72	4.11	-	-	-	-	-
Bicyclo[3.1.0]hex-2-ene, 2-methyl-5-(1-methylethyl)-	-	-	1.33	-	-	1.25	-	-	0.91	3.96	-	-	0.99	1.42	-	-	-	-	-	-	-	-	-
.alpha.-Pinene	-	-	-	-	4.12	2.06	406.12	4.72	7.80	12.07	2.39	-	5.97	-	-	-	-	-	-	7.09	-	-	-
Camphene	-	-	-	-	-	-	22.85	-	-	-	-	-	-	-	-	-	-	-	-	-	-	-	-
Bicyclo[3.1.1]heptane, 6,6-dimethyl-2-methylene-, (1S)-	-	-	-	-	-	-	543.45	-	-	-	-	-	2.67	2.67	-	-	-	-	-	-	-	-	-
.beta.-Myrcene	-	-	-	-	4.00	18.67	-	8.25	-	13.45	-	-	2.53	-	-	-	-	-	-	5.39	-	-	-
1-Decene	-	3.06	3.51	2.74	3.31	4.12	-	3.21	1.50	2.67	2.78	-	1.43	-	2.15	1.89	-	2.74	-	3.02	1.08	4.11	2.52
(+)-4-Carene	-	2.03	5.50	27.72	3.41	7.38	5.42	4.50	7.73	16.80	-	-	6.94	4.24	-	-	-	-	-	6.66	-	-	-
D-Limonene	5.96	63.74	85.84	105.95	39.96	36.53	201.63	66.15	67.15	84.91	16.43	17.93	50.03	50.69	27.49	1.55	46.55	11.17	12.54	55.47	2.89	4.20	4.50
.beta.-Ocimene	-	-	-	-	-	-	-	-	-	-	2.03	-	-	-	-	-	-	-	-	-	-	-	-
.gamma.-Terpinene	-	3.93	6.69	58.33	3.04	11.08	7.15	3.29	4.30	25.19	-	-	5.10	4.81	1.49	-	-	-	1.71	4.52	-	-	-
o-Isopropenyltoluene	4.51	4.29	6.35	5.45	74.56	-	13.42	185.82	209.16	89.54	24.09	-	90.06	5.49	-	-	-	-	2.63	123.81	-	4.74	-
Cyclohexene, 3-methyl-6-(1-methylethylidene)-	-	11.18	11.19	7.78	292.78	196.76	37.64	412.70	724.50	370.37	90.76	4.53	386.03	18.80	8.91	-	7.54	10.05	12.39	587.93	7.46	2.47	9.71
4-Undecene, (E)-	-	-	5.05	1.21	1.46	3.63	-	1.34	0.80	0.97	3.24	-	-	-	-	2.08	-	-	2.52	2.62	1.57	4.07	-
3-Tetradecene, (E)-	-	-	-	-	-	-	-	-	-	-	-	-	-	-	-	-	-	-	-	-	-	2.92	-
1,3,8-p-Menthatriene	-	-	-	-	7.33	-	-	28.51	22.30	11.90	-	-	9.41	-	-	-	-	-	-	12.64	-	2.46	-
4-Decene, 5-methyl-, (E)-	-	-	-	1.63	-	-	-	-	-	-	-	-	-	-	-	-	-	-	-	-	-	-	-
5-Undecene, (E)-	-	-	-	-	-	-	-	-	-	-	-	-	-	-	1.06	-	-	-	-	-	-	-	-
p-Mentha-1,5,8-triene	3.31	-	-	-	5.44	4.16	-	10.58	13.70	8.15	2.50	-	6.87	-	-	-	-	-	2.11	9.15	-	-	-
Bicyclo[3.2.2]non-8-en-6-ol, (1R,5-cis,6-cis)-	-	-	-	-	-	-	-	-	0.66	-	-	-	-	-	-	-	-	-	-	-	-	-	-
Bicyclo[2.2.1]hept-2-ene, 1,7,7-trimethyl-	-	-	-	-	-	-	-	-	-	-	-	1.26	-	-	-	2.06	-	1.34	-	-	-	-	-
.alpha.-Cubebene	-	-	-	-	-	-	-	1.29	-	-	-	-	-	11.77	4.43	-	-	15.01	4.90	4.43	6.08	3.34	6.39

1-Pentadecene	-	-	6.81	-	-	-	-	-	-	-	-	-	-	-	-	-	-	-	-	-	-	3.16	-
9-Octadecene, (E)-	-	7.96	-	-	-	-	-	-	-	-	2.21	-	-	-	-	-	-	-	-	-	-	-	-
.alfa.-Copaene	-	-	-	-	-	-	-	-	-	-	-	-	-	-	-	-	-	3.62	-	-	-	-	-
1-Tetradecene	-	2.29	-	1.65	2.16	-	-	2.38	-	1.88	-	-	-	-	-	-	-	-	-	2.70	-	-	1.74
Germacrene D	-	-	-	-	-	-	-	-	-	-	-	-	-	-	-	-	-	2.68	-	-	-	-	-
isoledene	-	-	-	-	-	-	-	1.01	-	-	-	-	-	-	-	-	-	-	-	-	0.99	-	-
.alpha.-Muurolene	-	-	-	-	-	-	-	-	-	-	-	-	-	-	-	-	-	6.65	-	-	-	-	-
cis-Calamenene	-	-	-	-	-	-	-	2.10	-	-	-	-	-	5.68	5.42	-	-	52.90	3.26	3.10	9.46	3.32	6.85
.gamma.-Muurolene	-	-	-	-	-	-	-	-	-	-	-	-	-	-	-	-	-	-	-	-	-	1.48	-
.alpha.-Calacorene	-	-	-	-	-	-	-	-	-	-	-	-	-	-	-	-	-	5.33	-	-	-	-	-
.alpha.-Corocalene	-	-	-	-	-	-	-	-	-	-	-	-	-	-	-	-	-	1.84	-	-	-	-	-
Pentadecane, 2,6,10,14-tetramethyl-	-	-	-	-	-	-	-	-	-	-	-	-	-	-	-	-	-	-	-	-	-	20.11	-
Aromatic hydrocarbons and their homologs																							
Toluene	5.99	2.38	2.48	14.47	12.35	1.31	33.40	1.21	-	4.10	1.98	6.61	-	38.54	9.44	1.84	6.58	6.72	1.86	1.26	-	0.82	-
o-Xylene	-	7.41	1.88	6.95	7.87	20.12	48.24	18.92	-	10.48	11.23	2.55	-	196.84	7.75	5.50	16.06	20.64	22.71	2.86	6.87	10.11	4.80
p-Xylene	-	-	-	-	-	39.14	-	-	-	-	-	-	-	-	-	-	-	-	-	4.04	-	-	-
Benzene, (1-methylethyl)-	-	-	-	-	-	-	-	-	-	-	-	-	-	2.64	-	-	-	-	-	-	-	-	-
p-Cymene	3.42	18.17	24.78	26.43	17.53	29.61	28.08	22.67	32.30	47.77	5.01	-	14.26	18.55	3.86	-	6.67	-	5.99	25.59	-	1.73	1.51
Benzene, n-butyl-	-	1.56	1.26	-	1.75	-	-	1.03	1.52	1.30	-	-	-	-	-	-	-	-	-	2.12	-	2.16	-
Benzene, 1-methyl-4-propyl-	-	-	2.11	-	1.74	1.49	-	0.75	-	1.56	-	-	-	-	1.44	-	-	-	1.38	1.70	-	1.05	-
Naphthalene, 1,2,3,4-tetrahydro-6-methyl-	-	-	-	-	-	-	-	-	-	-	-	-	-	-	-	-	-	-	-	-	-	1.42	-
Naphthalene, 1,2,3,4,4a,5,6,8a-octahydro-7-methyl-4-methylene-1-(1-methylethyl)-,	-	-	-	-	-	-	-	-	-	-	-	-	-	-	-	-	-	6.51	-	-	-	-	-

(1.alpha.,4a.beta.,8a.alpha.)-																							
Naphthalene, 1,2,4a,5,8,8a-hexahydro-4,7-dimethyl-1-(1-methylethyl)-, [1S-(1.alpha.,4a.beta.,8a.alpha.)]-	-	-	-	-	-	-	-	-	-	-	-	-	-	-	-	-	-	-	-	1.95	-	-	-
Phenol, 3,5-bis(1,1-dimethylethyl)-	9.94	2.34	2.81	2.48	2.16	2.20	-	2.16	1.93	1.97	2.73	1.80	1.37	10.85	2.69	1.45	9.45	2.52	2.20	2.11	2.07	19.68	2.48
Naphthalene, 1,2,3,4-tetrahydro-1,6-dimethyl-4-(1-methylethyl)-, (1S-cis)-	-	-	-	-	-	-	-	-	-	-	-	-	-	-	-	-	-	-	-	10.05	-	-	-
1-Isopropyl-4,7-dimethyl-1,2,3,5,6,8a-hexahydronaphthalene	-	-	-	-	-	-	-	-	-	-	-	-	-	-	-	-	-	-	-	4.17	-	-	-
Naphthalene, 1,2,3,4,4a,7-hexahydro-1,6-dimethyl-4-(1-methylethyl)-	-	-	-	-	-	-	-	-	-	-	-	-	-	-	-	-	-	12.63	-	2.72	2.52	-	-
Phenol, 2,6-bis(1,1-dimethylethyl)-4-(1-methylpropyl)-	-	-	-	-	-	-	-	-	-	-	-	-	-	-	-	-	-	-	-	-	-	59.02	-
Naphthalene, 1,6-dimethyl-4-(1-methylethyl)-	-	-	-	-	-	-	-	-	-	-	-	-	-	-	-	-	-	6.07	-	-	1.88	-	-
Others																							
Carbamic acid, monoammonium salt	11.45	7.70	7.82	9.95	7.56	6.95	18.21	7.21	7.66	8.67	11.78	4.42	5.52	28.61	9.21	4.88	13.33	6.53	9.47	6.36	5.08	14.10	10.60
Ethyl ether	-	0.39	0.28	1.96	1.14	0.94	-	0.50	0.82	0.82	1.66	0.65	0.79	9.33	1.24	-	-	0.72	0.34	0.88	0.71	1.12	0.69

Dimethyl sulfide	-	2.70	1.53	3.91	-	1.90	4.63	2.75	3.56	-	-	1.51	1.71	-	-	-	4.97	-	1.16	-	-	2.47	-
Ammonium acetate	-	-	-	-	-	-	-	-	-	-	-	-	-	-	-	-	3.77	-	-	-	-	-	-
Propanoic acid, anhydride	24.21	-	4.84	5.91	7.69	5.25	-	5.58	5.06	-	7.47	4.31	3.80	16.49	7.15	3.65	24.14	-	-	4.38	4.04	-	7.58
2H-Pyran, tetrahydro-2-methyl-	-	-	-	-	-	-	-	-	-	-	-	-	-	2.23	-	-	-	-	-	-	-	-	-
Oxime-, methoxy-phenyl-_	121.22	117.74	127.49	125.01	99.85	100.31	291.96	106.68	71.72	109.51	155.53	60.51	53.75	116.66	103.69	62.64	152.41	87.49	189.39	79.38	82.94	133.34	143.59
Anisole	-	-	-	-	-	-	-	1.80	-	-	-	-	-	-	-	-	-	-	-	-	-	-	-
Cedrol	-	-	-	-	-	-	-	-	-	-	-	-	-	-	-	-	-	-	-	-	-	18.44	-
Morpholine, 4-octadecyl-	-	-	-	-	-	-	-	-	-	-	-	-	-	-	-	-	-	-	-	-	-	20.09	-

[a] – means not detected.

Table S4. Total variance explained.

Principal component	Initial eigenvalue			Extraction sums of squared loadings		
	Total	% of variance	Cumulative %	Total	% of variance	Cumulative %
1	31.641	18.29	18.29	31.641	18.29	18.29
2	27.771	16.053	34.342	27.771	16.053	34.342
3	19.55	11.3	45.643	19.55	11.3	45.643
4	15.63	9.034	54.677	15.63	9.034	54.677
5	12.011	6.943	61.62	12.011	6.943	61.62
6	9.116	5.269	66.89	9.116	5.269	66.89
7	8.273	4.782	71.672	8.273	4.782	71.672
8	6.958	4.022	75.694	6.958	4.022	75.694
9	6.397	3.698	79.391	6.397	3.698	79.391
10	5.391	3.116	82.508	5.391	3.116	82.508
11	4.923	2.846	85.353	4.923	2.846	85.353
12	4.236	2.449	87.802	4.236	2.449	87.802
13	3.885	2.246	90.048	3.885	2.246	90.048
14	3.297	1.906	91.954	3.297	1.906	91.954
15	3.002	1.735	93.689	3.002	1.735	93.689
16	2.177	1.258	94.947	2.177	1.258	94.947
17	1.992	1.152	96.099	1.992	1.152	96.099
18	1.834	1.06	97.159	1.834	1.06	97.159
19	1.682	0.972	98.131	1.682	0.972	98.131
20	1.523	0.88	99.011	1.523	0.88	99.011
21	1.103	0.637	99.648	1.103	0.637	99.648
22	0.608	0.352	100.000			
